# *Mycobacterium tuberculosis* Exploits a Molecular *Off Switch* of the Immune System for Intracellular Survival

**DOI:** 10.1038/s41598-017-18528-y

**Published:** 2018-01-12

**Authors:** Ulrich von Both, Maurice Berk, Paul-Michael Agapow, Joseph D. Wright, Anna Git, Melissa Shea Hamilton, Greg Goldgof, Nazneen Siddiqui, Evangelos Bellos, Victoria J. Wright, Lachlan J. Coin, Sandra M. Newton, Michael Levin

**Affiliations:** 1 Section of Paediatric Infectious Diseases and Allergy, Department of Medicine, Imperial College London, London, UK; 2 Division of Paediatric Infectious Diseases, Dr von Hauner Children’s Hospital, University Hospital, Ludwig-Maximilians-University (LMU) Munich, Munich, Germany; 3 German Centre for Infection Research (DZIF), Partner Site Munich, Germany; 4 Cancer Research UK Cambridge Institute, Department of Oncology, University of Cambridge, Cambridge, UK; 5 Tuberculosis Research Centre, Respiratory Infections Centre, National Heart and Lung Institute, Imperial College London, London, UK; 6 Genomics of Development and Disease Division, Institute for Molecular Bioscience, The University of Queensland, Brisbane St Lucia, Australia; 7 Present Address: Department of Biochemistry, University of Cambridge, Cambridge, UK

## Abstract

*Mycobacterium tuberculosis* (*M. tuberculosis*) survives and multiplies inside human macrophages by subversion of immune mechanisms. Although these immune evasion strategies are well characterised functionally, the underlying molecular mechanisms are poorly understood. Here we show that during infection of human whole blood with *M. tuberculosis*, host gene transcriptional suppression, rather than activation, is the predominant response. Spatial, temporal and functional characterisation of repressed genes revealed their involvement in pathogen sensing and phagocytosis, degradation within the phagolysosome and antigen processing and presentation. To identify mechanisms underlying suppression of multiple immune genes we undertook epigenetic analyses. We identified significantly differentially expressed microRNAs with known targets in suppressed genes. In addition, after searching regions upstream of the start of transcription of suppressed genes for common sequence motifs, we discovered novel enriched composite sequence patterns, which corresponded to *Alu* repeat elements, transposable elements known to have wide ranging influences on gene expression. Our findings suggest that to survive within infected cells, mycobacteria exploit a complex immune “molecular off switch” controlled by both microRNAs and *Alu* regulatory elements.

## Introduction

**Figure 1:**
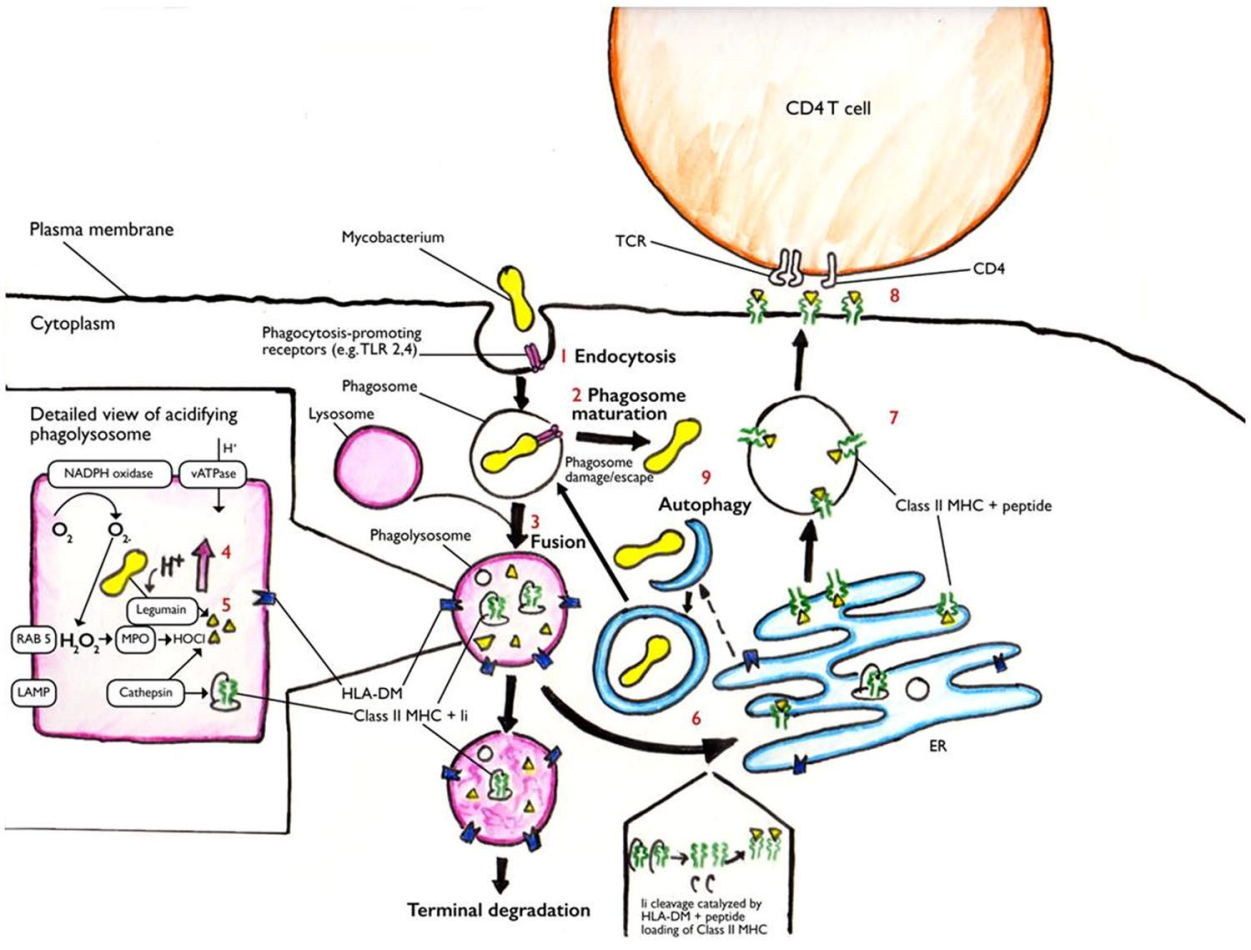
Intracellular mycobacteria evade host immunity. Cartoon showing various sites of immune subversion where mycobacteria are known to evade intracellular killing. References highlight key publications and relevant reviews showing impairment of steps 1-9 in *M. tuberculosis* infection. (**1**) Mycobacteria are recognized by phagocytosis-promoting receptors and engulfed within the early phagocytic vacuole^63–65^. (**2**) Maturation of the phagosome^66–68^. (**3**) Phagolysosome fusion and ingress of proteolytic enzymes^66,69–72^. (**4**) Acidification of phagocytic vacuole through activation of vATPase and NADPH-oxidase^66,70,73,74^. (**5**) Degradation of proteins to peptides^75^. (**6**) Cleavage of invariant chain (Ii) facilitated by HLA-DM and peptide-loading of class II MHC^76–78^. (**7**) Transport of class II MHC and peptide from the endoplasmic reticulum (ER) to the plasma membrane^77,79,80^. (**8**) Presentation of peptide antigen via class II MHC to CD4^+^T-cells^6,80,81^. (**9**) Free cytoplasmic bacteria are killed by autophagy and NLRP3 inflammasome activation^10,82^.

*Mycobacterium tuberculosis* (*M. tuberculosis*) currently infects a third of the world’s population, causing 9.6 million new cases of tuberculosis (TB) and 1.5 million deaths annually^1^. Following infection with *M. tuberculosis*, 90% of individuals successfully contain the pathogen but enter a state of long-term infection in which viable mycobacteria survive within infected cells and granulomata^2^. In approximately 10% of those infected, progression of infection occurs, resulting in active TB disease^3^. For the majority of patients with active TB, the disease appears to represent an immunological “stand-off”, where despite evidence of a vigorous host immune response involving both innate and acquired arms of the immune system, *M. tuberculosis* survives and multiplies, causing progressive destruction of lungs and other tissues. Mycobacteria are predominantly intracellular pathogens, and their ability to survive within human cells, despite evidence of an active host response, appears to be explained by their ability to subvert multiple components of the host immune response^4^ (Fig. 1). These include inhibition of pathogen sensing (Fig. 1(1)), phagosome maturation (Fig. 1(2)) and phagolysosome fusion (Fig. 1(3)), impairment of acidification of the phagocytic vacuole (Fig. 1(4)), impaired release and function of proteolytic enzymes in the phagocytic vacuole (Fig. 1(5)), inhibition of antigen processing and presentation and the impaired function of the MHC class II system which normally presents mycobacterial peptides to CD4^+^ T cells (Fig. 1(6–8))^5–8^. Furthermore, proteins released from mycobacteria (i.e. ESAT6 and CFP10), also enable the pathogen to escape from the phagolysosome (Fig. 1(9)), and *M. tuberculosis* infection impairs alternative intra-cytoplasmic killing via autophagy and NLRP3-inflammasome activation^9–11^.

Despite extensive documentation of the multiple functional defects in intracellular pathogen killing (Fig. 1), the underlying molecular mechanisms are largely unknown. Studies that describe molecular mechanisms have tended to focus on individual pathways in single cell types. To explore and characterise the molecular events occurring during interaction of human immune cells with *M. tuberculosis* in a more complex biological system, we infected whole blood from healthy volunteers with *M. tuberculosis* H37Rv and performed whole-genome RNA profiling over a 96 h time course. This well-validated infection model^12–14^ has advantages over isolated cells or non-human models, as it includes all the elements of the human immune response (phagocytes, T- and B- lymphocytes, plasma proteins including cytokines)^13^ and may be more revealing of the interplay of the cellular and molecular mechanisms involved in a complex biological infection system over time. Furthermore, the model has been shown to reflect changes in the immune response to mycobacteria, including differences between tuberculin-positive and -negative individuals^13^, changes induced by human immunodeficiency virus (HIV) infection and following antiretroviral treatment^15,16^, and changes in mycobacterial growth limitation induced by vaccines^17^.

## Results

We initially studied a “discovery” set of four healthy “mycobacteria-naïve” subjects (BCG-non-immunized, tuberculin skin test [TST]-negative) and validated the findings in a comparable second set of six volunteers. Fresh blood from each individual was inoculated with *M. tuberculosis* H37Rv and growth of mycobacteria was evaluated at 24 h intervals over a 96 h period, with concurrent analysis of genome-wide RNA, and expression of secreted and surface expressed proteins by enzyme linked immunosorbent assay (ELISA) and fluorescence activated cell sorting (FACS), respectively. To identify significant changes in gene transcription and determine direction (up or down) of differential expression over time we used a novel smoothing splines mixed-effects (SME) model^18^.

### *M. tuberculosis* infection of whole blood causes predominantly suppression (down-regulation) of both gene and protein expression

**Figure 2:**
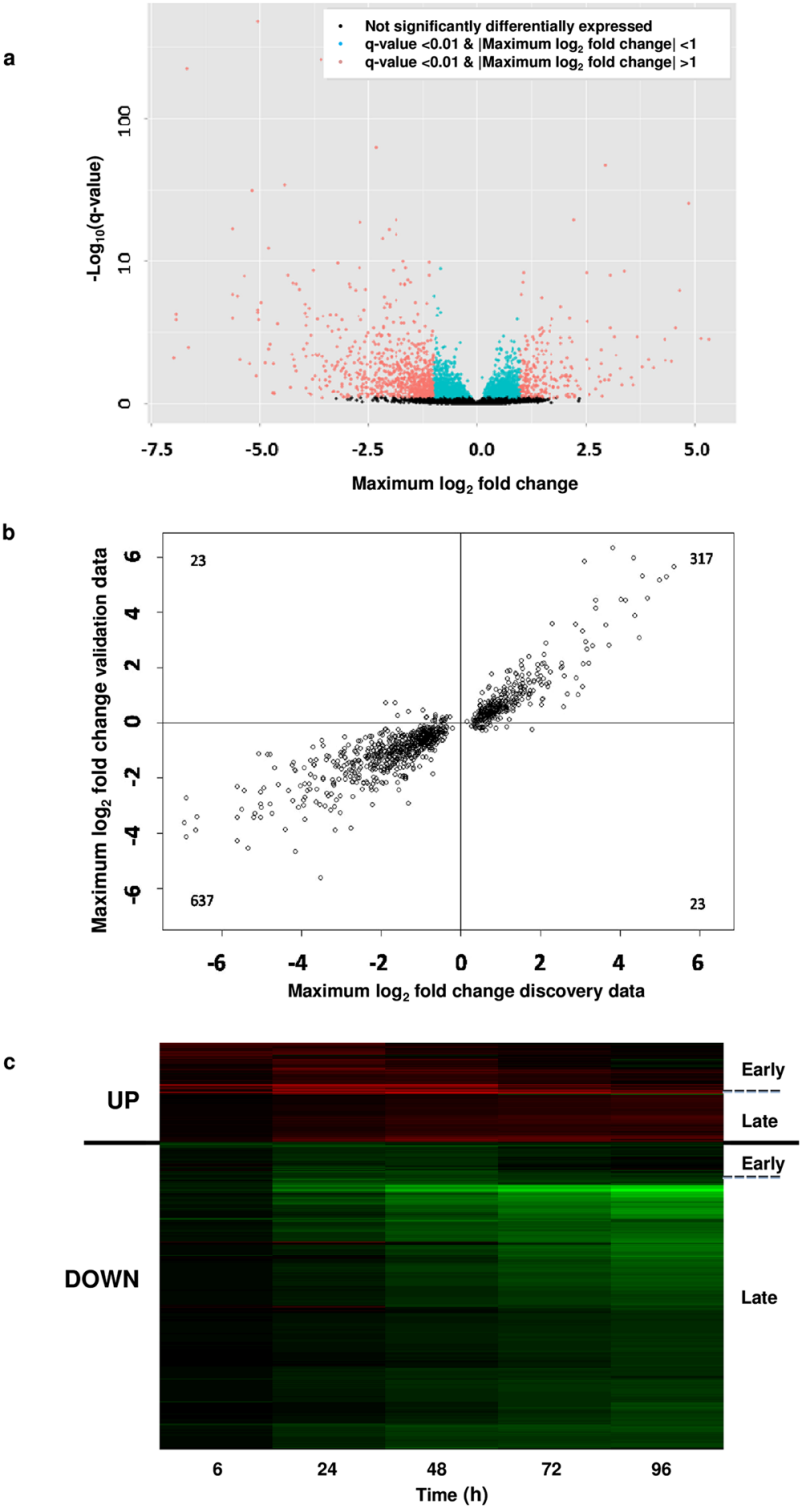
*M. tuberculosis* induces differentially expressed transcripts during *in vitro* infection of whole blood. (**a**) Volcano plot showing transcripts differentially expressed in response to infection of whole blood with *M. tuberculosis* over time (0–96 h) in the discovery data set (using blood from 4 individual donors). Blue dots (n = 2,858) represent significantly differentially expressed (SDE) transcripts between matched infected and uninfected samples, and with |maximum log_2_FC| < 1; red dots (n = 1,026) represent SDE transcripts with |maximum log_2_FC| > 1. (**b**) Validation of changes in mRNA expression; correlation of |maximum log_2_FC| between SDE transcripts in the discovery and validation study (top 1000 transcripts shown). (**c**) Heat map showing timing of maximum mRNA expression of up- (red) and down- (green) regulated genes for the top 1000 transcripts with |maximum log_2_FC| > 1 in *M. tuberculosis* infected whole blood as compared to uninfected whole blood. Black represents no difference in expression. The relative degree of transcript abundance is indicated by the colour intensity derived from the fitted mean expression levels over time (see methods).

The SME model is a novel and powerful method for statistical analysis of time-course data. After statistical correction for multiple comparisons (false discovery rate [FDR] q-value < 0.01), this SME model identified 3,884 probes (2,994 unique genes) in the discovery set as being significantly differentially expressed (SDE) during infection of human blood with *M. tuberculosis* over the 96 h time-course relative to the uninfected controls (Supplementary Table S1). Of all SDE probes having a log_2_ FC > 1 or <−1, 75% (766/1026) were suppressed (binominal proportion test p-value < 2.2 × 10^−16^) (Fig. 2a–c). Greater than 95% of the SDE probes in the discovery set displayed consistent direction of expression in both discovery and the validation cohorts (Fig. 2b). For validation of our findings and to confirm that differential RNA expression reflected changes at the protein level, we measured a selection of secreted and cell-associated proteins (TNF-α, IFN-γ, HLA-DM) by ELISA and FACS respectively, encoded by SDE transcripts. Protein expression paralleled the changes in gene expression (Supplementary Fig. S1).

### Cellular location, biological function and timing of gene expression

**Figure 3:**
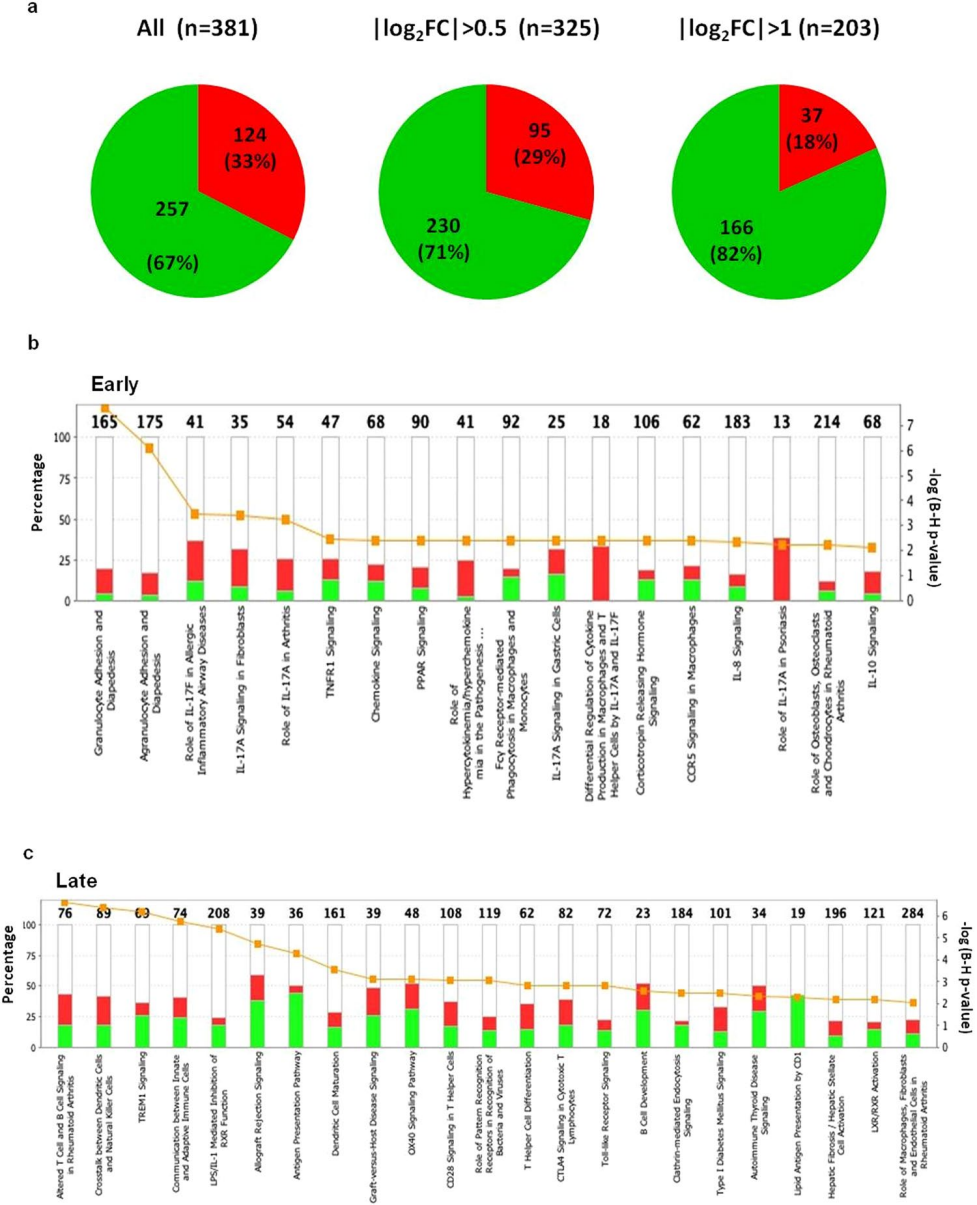
Balance of up- and down-regulation of significantly differentially expressed (SDE) genes in response *to M. tuberculosis* infection of whole blood over a 96 h time course. (**a**) Proportion of SDE genes coding for surface expressed proteins showing up- or down-regulation over 96 h. Red = up regulated genes; green = down-regulated genes (q < 0.001). Down-regulation is the predominant response to infection, proportion of down-regulated genes increases with effect size, represented by |log_2_FC|. (**b**) Assignment of early (0–48 h) and late (48–96 h) SDE genes to canonical pathways (IPA®) according to timing of infection. Height of individual bars corresponds to percentage relative to total number of genes (top of each bar) assigned to respective pathway. Red = up-regulated, green = down-regulated genes. Orange dots show the −log(B–H adjusted p-value) for each pathway.

To explore the biology of the transcriptional response we used functional databases (Ingenuity Pathway Analysis [IPA®] and KEGG pathways [http://www.genome.jp/kegg/pathway.html]) to assign SDE genes to functional categories, cellular location and canonical pathways. We found a striking predominance of down-regulated genes whose proteins are components of the cell surface (Fig. 3a). Of all 381 SDE genes coding for surface expressed proteins, including cellular recognition receptors, signalling molecules and integrins, 67% were down-regulated after *M. tuberculosis* infection over 96 h. When looking at genes with a max. log_2_ fold change (FC) < −1 and >1 (n = 203) the percentage of down-regulated genes increased (82%).

We observed differences in timing of maximal gene expression or suppression with some genes rapidly reaching their maximum FC (either up- or down-regulated) early, within the first 48 h. A second group reached maximum expression/repression in the 48–96 h time period (late) (Fig. 3b). These different temporal patterns of expression were further analysed by biological pathway and function. Early SDE genes were those largely involved in granulocyte and agranulocyte adhesion and diapedesis, activation of IL-17, peroxisome proliferator-activated receptor (PPAR) and TNFR1 signalling, FCϒ receptor-mediated phagocytosis in macrophages and monocytes, as well as chemokine CCR5, IL-8 and IL-10 signalling.

In contrast, late SDE genes were those involved in T- and B-cell signalling, communication between dendritic cells and natural killer cells, TREM1 signalling, communication between innate and adaptive immune cells and antigen processing and presentation. We also observed differences in the balance between up-regulation and down-regulation of genes according to timing. Increased transcript abundance predominated in the early time-points while genes less abundant became the predominant pattern at later time -points (Fig. 3b).

**Figure 4:**
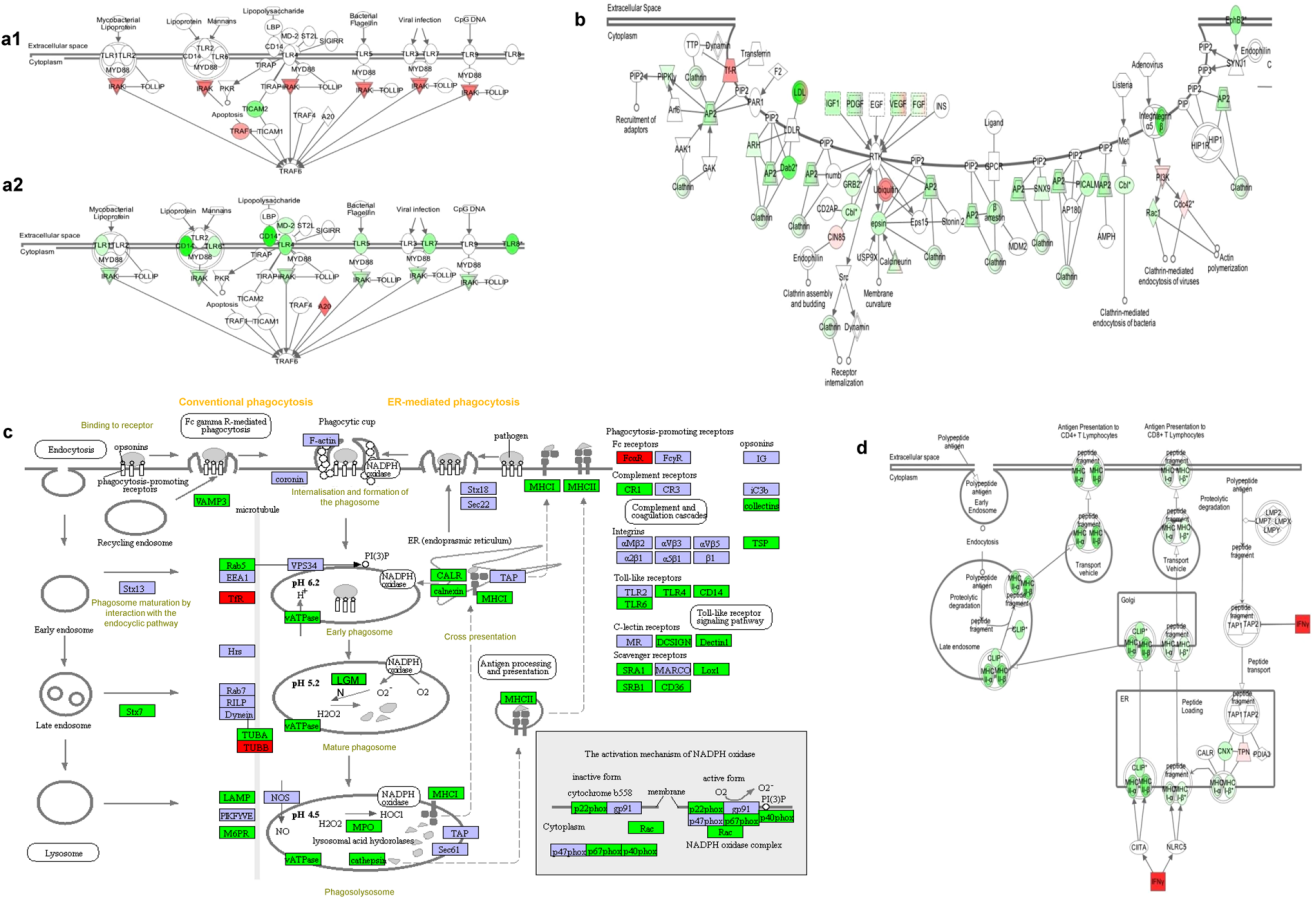
Expression of key biological pathways involved in *M. tuberculosis* recognition and intracellular killing. Significantly differentially expressed genes in response to *M. tuberculosis* infection of whole blood over 96 h, relative to uninfected whole blood controls, are up-regulated (coloured in red) or down-regulated (coloured in green). Intensity of colour indicates degree of transcript abundance. Genes coloured in blue did not reach significance. All pathways are generated using (IPA®) unless stated. (**a1**) Early (0–48 h) surface expressed receptors in the TLR pathway; (**a2**) Late (48–96 h) surface expressed receptors in the TLR pathway; (**b**) Clathrin-mediated endocytosis pathway (0–96 h); (**c**) Phagocytic vacuole pathway (using Kegg Pathway analysis, www.kegg.jp/kegg/^83–85^)- acidification and proteolytic digestion within the phagolysosome (0–96 h); (**d)** Antigen processing and presentation pathway (0–96 h).

The predominance of down-regulation of surface expressed molecules and repression of genes in key pathways known to be involved in mycobacterial immunity is highlighted by Toll-Like Receptor (TLR) (Fig. 4a1–2), pattern recognition receptor (Supplementary Fig. S2), and TREM1 signalling pathways (Supplementary Fig. S3). For each of these key signalling pathways the initial up-regulation (early 0–48 h) of signalling molecules such as *IRAK1* in the TLR signalling pathway (Fig. 4a1), *NF-κB*, *TNF-α* and *IL-6* in the pattern recognition receptor pathway or *TNF-α*, *IL-6*, *IL-8*, *MIP-1α* and *MCP-1* in the TREM1 signalling pathway is followed at the later time points by repression of multiple components including *TLR-1*, *TLR-4*, *TLR-6*, *IRAK1*, *CD14*, *DECTIN1*, *DAP12* and *CD11c*.

To determine that the increased proportion of down-regulated genes was not due to the presence of cell death, a trypan blue exclusion experiment on peripheral blood mononuclear cells (PBMC) and FACS analysis on whole blood was undertaken to assess viability of cells in the *M. tuberculosis* infected and uninfected samples over the 96 hour time-course. Whilst there was a 55% reduction in total PBMC over 96 h across all samples there was no significant difference between the uninfected and infected samples for each donor (n = 2) (Supplementary Table S2). Using FACS analysis there was a reduction (<15%) in total cell viability of peripheral blood leucocytes (PBLs) as a consequence of time rather than infection. At 96 h there was greater than 86% monocyte viability, and 100% lymphocyte viability in the infected compared to uninfected samples, however we did observe a marked reduction (78%) in the viability of short-lived granulocytes (CD8^+^ neutrophils) as a result of both time and infection (Supplementary Table S3). To confirm that the reduced RNA expression was not a consequence of the reduction in neutrophils or monocytes during the time course, we compared the direction of gene expression in our data set with data from Tailleux *et al*.^19^ who investigated RNA expression in isolated macrophages and dendritic cells infected with *M. tuberculosis*, at 48 h post-infection. We found 100% concordance of all SDE genes at this time-point that matched by name and direction of expression (down-regulation) in both data sets (69 out of 141 genes, 49%). (Supplementary Table S4). We also confirmed viability of mycobacteria in all infected samples (0–96 h) by colony forming unit (CFU) analysis.

### Genes involved in intracellular killing are repressed

To explore how gene repression or activation might relate to the functional defects in phagocytosis, phagolysosome fusion, acidification of the phagocytic vesicle and antigen processing and presentation observed in *M. tuberculosis* infection, we explored the pattern of gene expression or repression in these pathways. We found predominant down-regulation of gene expression in all stages of the phagocytosis and antigen presentation pathways over the 96 h time-course (Fig. 4b–d). The majority of genes of the clathrin-mediated endocytosis pathway and adapters required for the formation of clathrin coated membrane invagination were down-regulated, including multiple components of clathrin, epsin, calcineurin and β-arrestin as well as *DAB2*, *RAC*1, *SNX9* and PICALM. In addition, genes acting through the RTK receptor were repressed, including *IGF1*, *PDGF*, *VGEF* and *FGF* (Fig. 4b).

Enzyme systems required for acidification of the phagolysosome were down-regulated (Fig. 4c). The multi-component enzyme v-ATPase, which functions to pump hydrogen ions into the phagocytic vesicle, showed down-regulation of multiple components. Similarly there was marked repression of key components of the NADPH oxidase system required to generate reactive oxygen species, such as *RAC1*, *NCF2 (*also known as *p67PHOX)* or *NCF4* (also known as *p40PHOX*). The enzyme systems required to degrade proteins within the phagolysosome including myeloperoxidase (*MPO*), almost all variants of the group of serine/cysteine proteases (*cathepsin A, B, C, E, F, H, K, L1, L2* and *S*) and legumain (*LGMN*) were also suppressed. The antigen processing and presentation pathway showed down-regulation of multiple components, including *CLIP*, and *class I* and *II MHC α* and *β* (Fig. 4d). We also found down-regulation of genes in the NLRP3 inflammasome, including *NLRP3*, *PYCARD* (also known as *ASC*), *CARD6*, *CARD9* and *NLRC4*, while the process of autophagy was not significantly affected.

### MicroRNAs are significantly associated with gene expression during infection of whole blood with *M. tuberculosis*

**Figure 5:**
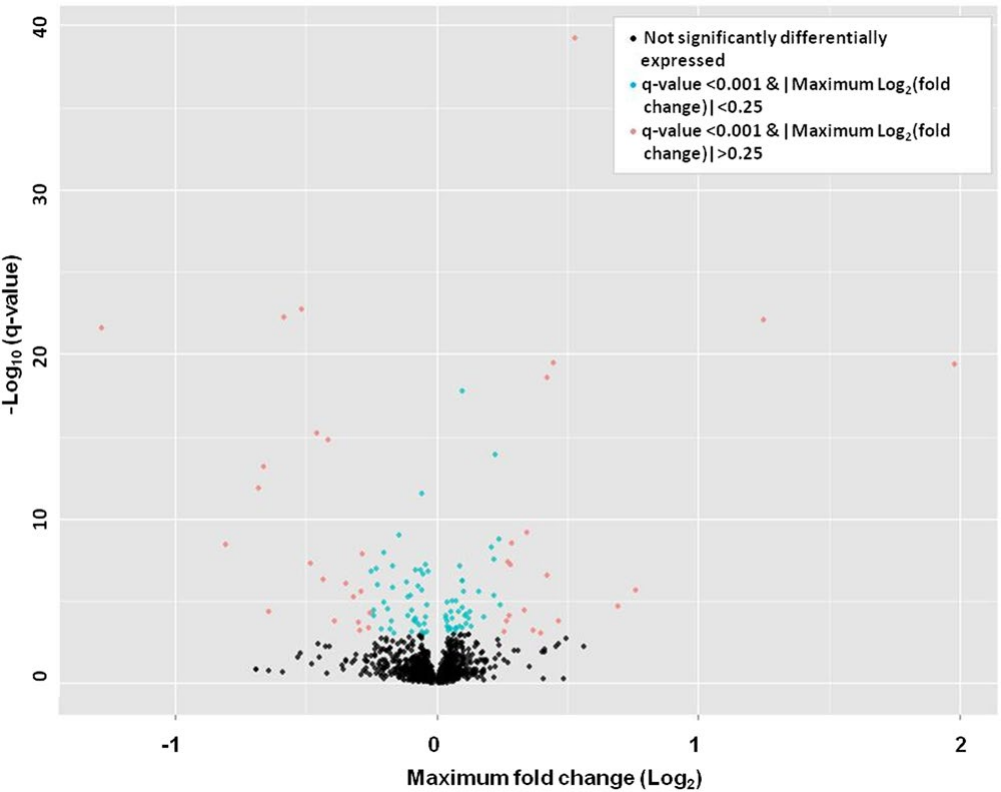
*M. tuberculosis* infection of whole blood induces differential expression of micro RNAs (miRNAs). Volcano plot showing differentially expressed miRNAs (probe level) in response to *M. tuberculosis* infection of whole blood over time (0–96 h, discovery set, using whole blood from 4 individual donors) in infected compared to uninfected, matched subjects. Black dots represent non-significant miRNA transcripts, blue dots (n = 71) SDE transcripts with a |maximum log_2_FC| < 0.25 and red dots (n = 50) SDE transcripts with a |maximum log_2_FC| > 0.25.

As microRNAs (miRNAs) have been implicated in gene regulation at the post-transcriptional level^20–22^, particularly in gene silencing, we studied the full 96 h time course of miRNA expression concurrently with mRNA expression in response to *M. tuberculosis* infection. We found 97 miRNAs SDE (q-value < 0.001, log_2_ FC > 0.25 or <−0.25) during the course of *M. tuberculosis* infection of whole blood (Fig. 5 and Supplementary Table S5). For these miRNAs we found a significant over-representation of mRNA targets amongst a) all SDE mRNA transcripts and b) all SDE down-regulated mRNA transcripts, using the *MicroRNA Target Filter* of IPA® followed by a hypergeometric test statistic; p-values 6.9 × 10^−31^ and 9 × 10^−13^ for all SDE mRNA transcripts, and all SDE down-regulated mRNA transcripts respectively. These analyses demonstrated a significant association between the SDE miRNAs (both up- and down-regulated) and the down-regulated SDE mRNA transcripts in our whole blood mycobacterial infection model.

### Down-regulation of genes is not due to changes in DNA methylation

To investigate whether DNA-methylation might underlie the differences in gene expression, we studied DNA methylation over time in the same experimental conditions as used for the RNA expression analysis, using Illumina Infinium Human Methylation 450 bead arrays. We did not detect any significant changes in DNA methylation between up- and down-regulated genes over the whole 96 h time-course (Supplementary Fig. S4).

### Down-regulated genes have common upstream regulatory elements

**Figure 6:**
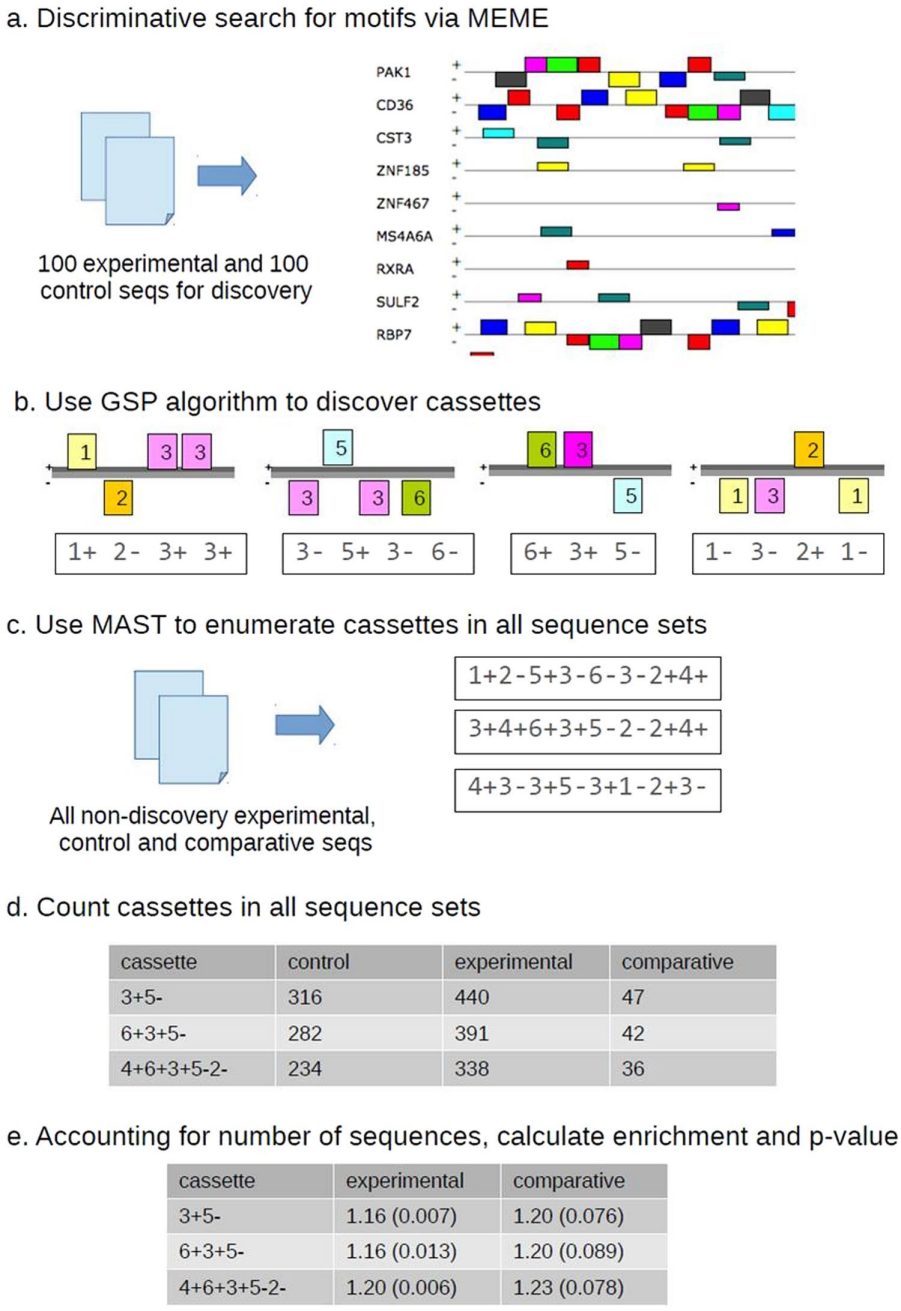
The workflow for motif cassette discovery and validation. (**a**) 100 experimental (down-regulated) and control (up-regulated) sequences are used for *de novo* motif discovery via MEME. (**b**) The sequences of motifs are reduced to strings of tokens and used by the modified general sequence pattern (GSP) algorithm to find cassettes of composite patterns. (**c**) The MAST program is used to find the motifs in non-discovery sets of experimental, control and comparative sequences. (**d**) These motif results are mined for the cassette patterns discovered above. (**e**) The enrichment of the cassettes in comparison to the control set is calculated, as is a *p*-value. *Note: the values shown are only for illustration purposes*.

Finally, we investigated the hypothesis that down-regulated genes were co-regulated, being supressed by common upstream regulatory regions or elements. We therefore contrasted common sequence motifs in the 1500 base pairs (bp) upstream of the transcription start site (TSS) of the 100 most significantly down- and up-regulated genes. No significant difference in distribution of transcription factor binding sites (TFBS) in regions upstream of down- vs. up-regulated genes was detected using OPOSSUM^23^. We next conducted a *de novo* search for motifs that were present more frequently upstream of the down-regulated than up-regulated genes. Using the heuristic motif searching tool ‘MEME’ to compare the above sequence datasets, 8 conserved motifs were identified upstream of down-regulated genes exclusively, ranging from 26–36 bp (Fig. 6). Further examination of these motifs showed that many were occurring in regular composite patterns, subsequently referred to as “cassettes”. These cassettes were composed of motifs found on either strand or in either orientation.

In order to establish frequency and distribution of the different patterns of “cassettes” in all up- and down-regulated genes, a modified general sequence pattern (GSP)^24,25^ algorithm was used to recognise and enumerate these cassettes (Supplementary Fig. S5). A number of cassettes of varying lengths were discovered, occurring in 20–40% of the examined sequences upstream of the down-regulated genes. The relationship between the cassettes is shown in Supplementary Fig. S6.

### Cassette enumeration in sequence datasets and identification of *Alu* repeat elements

Subsequently, we counted the wider occurrence of the cassettes identified. This was performed in the equivalent upstream regions of the down- and up-regulated genes that were not used for discovering the motifs (1,312 and 1,093 respectively) and for comparison from other gene datasets: 797 genes whose expression was unchanged by *M. tuberculosis* infection, and a subset of 51 down-regulated genes that were identified as associated with the phagosome/phagolysosome using KEGG and IPA® pathways (Supplementary Table S6). The MAST tool (part of MEME suite) was used to search for the motifs discovered in the initial discovery step, and then these patterns of motifs were searched for the cassettes found above. The enrichment (i.e. relative frequency) of each cassette within each dataset was calculated as the frequency of the cassette compared to the control (up-regulated) dataset. A p-value was created for the enrichment. We found significant enrichment of each individual cassette in the down-regulated genes relative to both the up-regulated and unchanged genes (Table 1). We also observed significant enrichment of 10 out of 19 cassettes within a list of 51 SDE down-regulated genes (q < 0.01) annotated to the phagosome-signalling pathway (Table 1 and Supplementary Table S7).

**Table 1:** Enrichment of cassettes upstream of different gene sets.

Cassette	Experimental^a^		Unchanged^b^		Phagosome-associated	
	Enrichment	p-value	Enrichment	p-value	Enrichment	p-value
3+5−	1.159	***7.49E−03***	0.942	7.83E−01	1.419	***3.90E−02***
3−2+	1.178	***1.98E−03***	0.978	6.15E−01	1.475	***1.90E−02***
3−6−	1.107	***3.82E−02***	0.931	8.41E−01	1.365	***4.77E−02***
5−2−	1.168	***5.22E−03***	0.958	7.12E−01	1.335	7.35E−02
6−4−	1.117	***2.71E−02***	0.946	7.79E−01	1.3	8.13E−02
8−4+	1.138	***1.08E−02***	0.953	7.49E−01	1.437	***2.49E−02***
3−2+5+	1.196	***2.73E−03***	0.978	6.02E−01	1.372	6.69E−02
3−6−4−	1.113	***3.99E−02***	0.891	9.33E−01	1.415	***4.01E−02***
6+3+5−	1.157	***1.33E−02***	0.909	8.73E−01	1.292	1.12E−01
6−4−8+	1.155	***1.01E−02***	0.947	7.56E−01	1.369	6.01E−02
3−6−4−8+	1.144	***2.15E−02***	0.888	9.22E−01	1.496	***2.89E−02***
4+6+3+5−	1.17	***1.12E−02***	0.888	9.13E−01	1.38	7.21E−02
4+6+3+5−2−	1.202	***6.25E−03***	0.897	8.76E−01	1.557	***2.98E−02***
8−4+6+3+5−	1.184	***1.04E−02***	0.849	9.59E−01	1.407	7.12E−02
4+6+3+5−2−3+	1.253	***2.09E−03***	0.943	7.19E−01	1.617	***2.72E−02***
8−4+6+3+5−2−	1.219	***5.86E−03***	0.858	9.35E−01	1.594	***3.02E−02***
1+8−4+6+3+5−2−	1.251	***7.55E−03***	0.903	8.05E−01	1.393	1.16E−01
8−4+6+3+5−2−3+	1.253	***3.56E−03***	0.897	8.42E−01	1.64	***3.00E−02***
1+8−4+6+3+5−2−3+	1.306	***3.02E−03***	0.924	7.34E−01	1.362	1.40E−01

Phagosome-associated genes are from the experimental data that were associated with the respective function or compartment based on KEGG pathways. Enrichment is calculated relative to the proportion of sequences containing at least one of the respective cassettes, relative to the control (non-discovery up-regulated) set. The *p*-value of the enrichment is calculated using the beta-binomial. *P*-values less than 0.05 are in bold. ^a^Experimental dataset (non-discovery down-regulated gene sequences), ^b^Unchanged dataset (sequences of genes without differential expression).

“Exemplar” sequences for each cassette were identified by finding the three cassette sequences with the most significant p-value (calculated by combining the p-value of each constituent motif) and extracting the associated sequence. BLAST analysis of the respective cassette sequences against the NCBI REFSeq database, identified human *Alu* elements as close matches which were then directly confirmed using the “human *Alu* elements” tool within the blastn suite (Supplementary Table S8). The exemplar cassettes were matched to the *Alu S* family, specifically the *Alu Sb* or *Alu Sx* sub-types.

### Other intracellular pathogens are associated with a similar host immune transcriptional response

In order to establish whether the down-regulation of genes we observed is also seen when whole blood is infected with non-pathogenic mycobacteria, we studied RNA expression in response to *M. bovis* BCG, using the same experimental methods as described for *M. tuberculosis*. We found that 88% of genes showing significant differential expression in response to *M. tuberculosis* infection followed the same direction of gene regulation (up or down) when whole blood was infected with *M. bovis* BCG (highly significant correlation, p-value 2.2 × 10^−16^) (See Supplementary Fig. S7).

To investigate whether the down-regulation of key genes we observed following infection of human cells with *M. tuberculosis* and *M. bovis* BCG also occurs in response to other intracellular pathogens, we compared the pattern of RNA expression in our *M. tuberculosis* data with the reported RNA expression changes over 72 h in a human macrophage cell line infected with *Leishmania major* or *Leishmania amazonensis*^26^. As the *in vitro* infection experiment and time course of the Leishmania study were similar to that in our study, we were able to directly compare the log_2_ FC of the 1000 most SDE genes in response to *M. tuberculosis* with the log_2_ FC of the same genes in Leishmania infection.

Again, we observed a remarkable concordance of the direction of expression of host genes in response to *M. tuberculosis* with the genes SDE in response to both species of Leishmania (Supplementary Fig. S8 and Supplementary Tables S9 and S10). A Pearson correlation coefficient calculated for the respective datasets revealed a highly significant correlation: *M. tuberculosis* and *L. major* log_2_ FC: Pearson’s r = 56.6% (p-value 2.2 × 10^−16^), and *M. tuberculosis* and *L. amazonensis* log_2_ FC: Pearson’s r = 60.5% (p-value 2.1 × 10^−16^). The highly significant correlation between the SDE host genes in response to *M. tuberculosis*, *M. bovis* BCG and both species of Leishmania, suggests common mechanisms may be involved in the genomic response to different intracellular pathogens.

## Discussion

We used a well-validated whole blood infection model and a novel statistical method for RNA expression analysis over a time course to explore the human host response to *M. tuberculosis* infection. This SME model enables the expression of each individual gene over time to be calculated, allowing for an accurate and dynamic illustration of the transcriptional changes in infected versus uninfected samples.

We show that the predominant genomic response to *M. tuberculosis* infection of human whole blood is down-regulation of genes; 75% of the SDE genes (|maximum log_2_ FC| > 1) were suppressed. The dynamic and temporal pattern of gene expression revealed initial up-regulation of genes coding for pro-inflammatory cytokines followed by a later repression of a wide range of cell surface receptors required for inter-cell communication, and a down-regulation of genes in the key pathways required for intracellular killing of mycobacteria.

Profound and consistent down-regulation of genes involved in phagolysosome formation was observed, including the endocytic process, which is the first step in entrapment of mycobacteria within the phagolysosome. Most components of the clathrin-mediated endocytosis pathway were suppressed, a pathway that had recently been reported in the context of interaction with the *M. tuberculosis* Mtbhsp60^27^. In addition, key components of the enzyme systems required for acidification of the phagocytic vesicle (v-ATPase complex) and the generation of reactive oxygen mediated species and the phagocytic burst (NADPH-oxidase complex) were also significantly suppressed. Key enzymes involved in degradation of proteins within the phagolysosome were down-regulated including myeloperoxidase (*MPO*), legumain (*LGMN*) and almost all variants of cathepsins, as were the class I and II MHC α and β components of the antigen processing system leading to presentation of peptide within the context of MHC class II. The findings of suppression of the group of cathepsins are in agreement with a recent study of Pires *et al*., with a single exception of the CtsF gene which has been shown as activated in their study using monocyte-derived macrophages infected with *M. tuberculosis*^28^. Alternative pathways for killing and containment of mycobacteria outside the phagosome, including generation of the NLRP3 inflammasome showed similar repression of key genes.

There is always a concern that down-regulation of genes during *in vitro* cell culture may be caused by cell death. To address this issue we undertook analyses including trypan blue exclusion, FACS analysis of viable cells and comparative gene expression analysis with additional datasets from infection of isolated macrophages^19^. Whilst we observed a reduction in monocyte and neutrophil cell numbers over time, we also observed up-regulation of predominantly macrophage-expressed genes *(CCL22*, *IL-6*, *IL-1A*, *IL-1B* and *IL-23A*). Furthermore the modest reduction in cell numbers was insufficient to explain the large log fold change in gene expression that we observed.

Confirmation that the differential expression of mRNA transcripts is associated with protein expression and function was found in the close correlation between mRNA expression and protein measurements of inflammatory proteins in plasma such as TNF-α and IFN-γ or the cellular expression of HLA-DM detected by FACS. Whilst differences in the direction of SDE genes such as *NLRP3*^29^ and those in the clathrin mediated pathway^27^ have been reported in other *in vitro* infection studies, and *in vivo* whole blood samples from patients with tuberculosis, differences in experimental conditions (*in vivo* versus *in vitro* infection and sample types; whole blood/single cell types; time-points/time-course analysis) are likely to result in different responses in gene expression changes and direction in some reported studies.

Our analysis suggests an explanation of how the previously observed functional defects in intracellular killing of *M. tuberculosis* might be caused at the genomic level, and links previous findings of molecular defects in individual biological pathways together. We have shown that the initial genomic response to infection of human cells with *M. tuberculosis* is a short-lived burst with increased production of mRNA transcripts coding for inflammatory cytokines, including *TNF-α*, *IL-6*, *IL-8*, *MIP-1α* and *MCP-1*. However, once mycobacteria have entered the phagocytic cell, the genomic response appears to be down-regulation of genes coding for cell surface receptors including TLRs required to recognise infected cells, and down-regulation of the key pathways required for intracellular killing and processing and presentation of antigens to the T- and B-cells of the acquired arm of the immune system. Although functional defects in phagolysosome fusion, acidification and antigen processing and presentation have been well established in *M. tuberculosis* infection^5,7,8,30^, our study is the first to suggest that a key mechanism responsible for impaired function of these inflammatory pathways might be repression of genes coding for production of proteins in each of these pathways.

Our finding that down-regulation of immune gene transcription is a predominant response to *M. tuberculosis* infection raises the question of how repression or inactivation of such a large number of genes is mediated. The silencing of a very large number of genes (more than half of the response) may be mediated by a common epigenetic event rather than through production of specific transcription factors^22^. Since miRNAs have been shown to regulate expression levels of target genes, we explored the relationship between mRNA and miRNA expression. A high proportion of the genes that were repressed in response to mycobacterial infection are known targets for miRNAs showing significant differential expression in our experiment suggesting that the observed down-regulation of multiple genes may in part be due to miRNA-mediated regulatory processes. Some SDE miRNAs, including hsa-miR-155, hsa-miR-223 or hsa-miR-132 have already been highlighted by various studies in the context of immune regulation and pathogenesis of *M. tuberculosis* infection. For example, *CXCL2*, *CCL3*, and *IL-6* have been identified as direct targets of hsa-miR-223; hsa-miR-155 has been shown to modulate the function of *SHIP1*- a negative regulator of the *PI3K/Akt* pathway, and stimulation of monocytes with lipopolysaccharide (LPS) induced expression of hsa-miR-132^31^, and hsa-miR-155^20,21,32^. In addition, hsa-miR-582-5p^33^, hsa-miR-99b^34^, hsa-miR-125a^35^, hsa-miR-26a^31^ and hsa-miR-32* have also been shown to be affected in their respective expression in the context of *M. tuberculosis* infection. Still, some key miRNAs, such as hsa-miR-142-3p, were not SDE in our experiment, although hsa-miR-142-3p has been reported by Bettencourt *et al*.^36^ as being involved in phagocytosis of human macrophages. Differences in experimental conditions in whole blood models compared to single cell models are likely to result in different responses in expression changes to certain miRNAs.

Our findings are supported by another recent publication by Brace *et al*.^37^ showing that *M. tuberculosis* specifically targets negative regulatory pathways to augment immunopathology in macrophages following *in vitro* infection with *M. tuberculosis* H37Rv. The authors found global suppression of the MMP-inhibitory pathways in macrophages that were attributed to miRNA-mRNA interaction. Of note, two key miRNAs identified by Brace *et al*.^37^ (hsa-miR-22 and hsa-miR-199a) have also been found to be SDE in our data, further supporting the potential role of miRNAs in the down-regulatory effects we describe.

In order to explore the possibility that genes showing common patterns of regulation might have common upstream regulatory elements, we searched the upstream regions of the differentially expressed genes and identified a series of strongly conserved repeating patterns or “cassettes” significantly enriched in upstream regions of down-regulated genes. These common cassettes were subsequently identified as *Alu* elements. We found that key genes annotated to the phagosome-signalling pathway and significantly repressed in response to *M. tuberculosis* infection of whole blood, showed significant enrichment for these “cassettes” suggesting that this gene regulatory effect plays an important role in disarming key steps of the phagosome pathway. These *Alu* elements are the most abundant transposable elements in the human genome, comprising more than a million copies and more than 10% of all human genomic sequences^38^. As a short interspersed element (SINE), *Alu* is approximately 300 bp in length consisting of two similar monomers with characteristic linker and flanking sequences. *Alu* elements are grouped by sequence into several broad families, which are believed to roughly reflect their evolution and spread throughout the genome^39,40^. *Alu* elements contain active (i.e transposable) which are believed to have originated about 30 million years ago, after the split of the old and new world monkeys^41^. *Alu* elements themselves do not encode proteins but contain binding and promotion sites for RNA polymerase III, and thus can potentially produce *Alu* mRNA, although the level of expression is highly dependent upon the genomic elements surrounding the *Alu* elements, epigenetic silencing by the host genome, and progressive erosion of the *Alu* sequence by mutation.

A parasitic genetic element can interfere with gene regulation by the simple act of insertion into a coding region^42^, but *Alu* elements appear to have been coopted into a variety of regulatory roles by the human genome^43,44^. These include polyadenylation, splicing, and ADAR (adenosine deaminase acting on RNA) editing. Levels of *Alu* mRNA increase during cellular insult, possibly due to chromatin opening and SINE mRNAs have been shown to control the heat shock response and attenuate of transcription by binding RNA polymerase II.

The significant enrichment of *Alu* elements observed in our study, which we suggest is involved in regulating transcriptional responses in human whole blood infected with *M. tuberculosis*, has also in parallel been observed in *M. tuberculosis* infected macrophages by Bouttier *et al*.^45^. Although our experimental set-up is a step closer to *in vivo* infection, their study used Chipseq analysis of H3K4 monomethylation (H3K4me1) in THP-1 cells infected with *M. tuberculosis* H37Rv for 24 h and identified regions (40%) that contained *Alu* S and J repeat elements. Examination of these *Alu* elements revealed transcription factor binding sites (TFBS) implicated in macrophage differentiation, survival and, response to stress and infection. In addition, genes proximal to H3K4me-1-associated *Alu* repeats were enriched for loci implicated in human innate immune defense and cell death pathways in *M. tuberculosis* infection^45^. *Alu* elements have long been known for harboring TFBS^46^. Though we identified *Alu* elements significantly enriched in the upstream regions of down-regulated genes, there was no significant difference in the distribution of TFBS between down- and up-regulated genes. This may support the hypothesis that it is the presence of *Alu* elements rather than TFBS that play an important role in down-regulating a large number of genes. While Bouttier *et al*.^45^ conducted their investigation using an isolated cell line and a predefined subset of genes, we used human whole blood and followed a genome-wide RNA analysis starting from the observation of a profound down regulation of genes, subsequently analyzing the subset showing the most significant down regulation. Thus, in the context of a growing body of literature on the regulatory roles of “selfish” genetic elements, the sequences we have identified as *Alu* elements may be coopted as *upstream gene regulatory elements* that control the down-regulation of genes involved in immune response to mycobacteria – an effective *off-switch* mechanism promoting the intracellular survival of *M. tuberculosis* in the human host. Although further investigation of the mechanisms underlying the association of the *Alu* elements with the gene expression changes we have observed is needed, it is also possible that other long-range regulatory mechanisms remain to be identified as we have only searched the region immediately upstream of each gene promoter.

The human immune system is remarkable in the speed in which it responds to the microbial pathogens to which we are all exposed throughout life. This inflammatory response successfully eliminates most invading pathogens, or localizes them to the site of infection until the cellular and antibody responses of the acquired immune system develop. Despite the evolutionary success of the human immune and inflammatory system in ensuring our survival in the face of the myriad of microbes to which we are exposed, excessive or uncontrolled inflammation is associated with severe illness, organ dysfunction and death, as occurs in conditions such as systemic inflammatory response syndrome or septic shock^47^. It is, therefore, not surprising that the diversity of the inflammatory responses to pathogens have been matched by an equally varied and powerful set of biological responses which have evolved to down-regulate the inflammatory process and localise inflammation at the site of infection. The two systems: activation of inflammation and down-regulation of the inflammatory response exist in a carefully balanced “yin/yang“-duality which functions to prevent either excessive inflammation or ineffective immune responses - both of which can be damaging to the host.

We hypothesise that the widespread suppression of gene transcription, particularly those involving key inflammatory pathways, which we have observed in response to *M. tuberculosis* infection, may indicate the function of a molecular “*off switch*” within the immune system which is activated to prevent excessive or uncontrolled inflammation. Silencing of immune response genes may not be unique to *M. tuberculosis* infection but may represent a common mechanism for down-regulating excessive inflammatory responses in many other infections. This hypothesis is supported by our finding that gene expression in whole blood infected with *M. bovis* BCG, is remarkably similar to that observed with *M. tuberculosis* infection, with a predominance of down-regulated genes in intracellular killing pathways. Furthermore, our comparative analysis of the host transcriptomic response to two different Leishmania species during *in vitro* infection of a human macrophage cell line supports this hypothesis. A highly significant correlation was demonstrated between the SDE genes in the Leishmania infection data sets and those in *M. tuberculosis* and *M. bovis* BCG.

Although down-regulation of inflammation, through the regulatory elements upstream of specific groups of genes described here, may be common to many infections, the suppression of genes and pathways essential for containment of mycobacteria (Fig. 1) may have resulted in an immunological “window” in the immune response which is exploited by intracellular pathogens. We suggest that mycobacteria, and potentially other intracellular pathogens, may have evolved to exploit the *common* “*molecular off switch*” of the immune system, which is responsible for down-regulating inflammation, to enable their intracellular survival. Our data suggests that the functioning of this “*off switch*” is in part mediated by both miRNAs and *Alu* elements upstream of the suppressed genes.

## Materials and Methods

### Mycobacterial strains

All *M. tuberculosis* culture and infection work was undertaken in the Section of Paediatric Diseases, Imperial College London in a Containment Level 3 laboratory adhering to Imperial College London safety practices and policies. All *M. bovis* BCG work was undertaken at the same institute within a Containment Level 2 laboratory. All class 2 and class 3 mycobacterial work has been notified to the Health and Safety Executive (HSE) and consent to work with such organisms has been approved.

*M. tuberculosis* H37Rv (*M. tuberculosis*) was obtained from the American Type Culture Collection (ATCC, U.S.A). *M. bovis* BCG *lux* (BCG, Montreal strain) obtained from Professor Young’s lab (Imperial College London) was transformed with the reporter plasmid construct pSMT1 as previously described in detail elsewhere^12^.

### Mycobacterial growth conditions

*M. tuberculosis* and *M. bovis BCG* were grown in a shaking incubator at 37 °C until mid-log phase in Middlebrook 7H9 broth (Difco, MI) medium containing 0.2% glycerol (Sigma-Aldrich), 0.05% tween 80 (Sigma-Aldrich), 10% ADC enrichment (Difco, MI). 50 ug/ml hygromycin (Roche) was added to *M. bovis* BCG cultures as antibiotic selection marker. Aliquots were frozen in 15% glycerol and stored at −80 °C. The number of colony forming units (CFU)/ml was determined by serial dilution on 7H11 agar (Difco, Detroit, MI) containing 0.5% glycerol, 10% oleic acid-albumin-dextrose-catalase (Difco, MI) enrichment. Prior to infection assays, a vial of mycobacteria was defrosted and grown to mid log phase. Cultures were centrifuged at 3200 rpm for 10 minutes, supernatant decanted and the cell pellet was resuspended in phosphate buffered saline (PBS) (Sigma-Aldrich) and further diluted in PBS to the required mycobacterial inoculum.

### Human subjects

Ethical permission for this study was granted by the Local Research Ethics committee - Queen Square (Ref no 11/LO/0823). All healthy adult blood donors gave written informed consent to participate in this study. All personal data remains confidential via anonymisation and all results have been de-identified. All blood sampling and experiments were in accordance with the relevant guidelines and regulations.

Twenty millilitres of peripheral whole blood was taken from healthy adult donors recruited at Imperial College London (Discovery set, *n* = 4; Validation set, *n* = 6 for *M. tuberculosis* infection experiments; discovery set *n* = 4; validation set *n* = 5 for *M. bovis* BCG infection experiments). Donors ranged in age from 27–54 (mean 37 years) and all were BCG non-vaccinated, purified protein derivative (PPD) negative, and ELISPOT negative to standard mycobacterial antigens. Whole blood was diluted 1:1 in RPMI (Sigma-Aldrich) and 2 ml aliquots were dispensed per bijou tube.

### Whole blood mycobacterial infection assays

We used a modified version of a well-established whole blood mycobacterial infection model^13,17^. In total, two different infection assays were performed corresponding to a discovery and validation assay set for both *M. tuberculosis* and *M. bovis* BCG. In brief, mycobacteria were inoculated into the 2 ml aliquots of diluted whole blood samples to give a final inoculum of 1 CFU: 1 monocyte (multiplicity of infection [MOI] 1:1) assuming an average of 2 × 10^5^ monocytes/ml of diluted whole blood. Uninfected whole blood samples served as matched controls at each time point. In each experiment, all samples (discovery: *n* = 4 donors; validation: *n* = 6 donors for *M. tuberculosis;* discovery set *n* = 4; validation set *n* = 5 for *M. bovis* BCG infection experiments) were incubated for up to 96 h at 37 °C on a rocking shaker at 25 rev/min. At sequential time points (Discovery set 0, 6, 24, 48, 72, 96 h; Validation set 0, 24, 48, 72, 96 h), Trizol LS (Invitrogen) was added (3:1) to each infected/uninfected whole blood sample, samples inverted and stored at −80 °C until subsequent RNA extraction.

### Viability and growth control of mycobacteria in whole blood samples

Viability and growth of mycobacteria (measured as colony forming unit [CFU]) in whole blood samples was assessed in duplicate at 0 and 96 h by plating onto 7H11 agar containing 10% OADC enrichment in triplicate and incubating at 37 °C for 14 days. Growth was determined by calculating numbers of CFU at *T96*/*T0*.

### ELISA

Simultaneously infected and uninfected whole blood aliquots (duplicate) corresponding to each donor were spun at 3200 rpm for 10 min as described above, supernatants harvested and stored at −20 °C until assayed. TNF-α, and IFN-γ production was measured in the supernatants in duplicate at 24 h intervals for up to 96 h, using commercially available Eli-pairs (BD Biosciences, USA) according to manufacturer’s recommendations. All data quoted are median values.

### Fluorescent activating cell sorting (FACS) analysis

Whole blood was lysed by diluting in lysis buffer (1x) (Biolegend) and washed in cold FACS buffer to retrieve a leukocyte pellet. Cell populations were distinguished by fluorescently labelling using a previously optimised polychromatic staining panel; LIVE/DEAD® Fixable Blue Dead Cell Stain Kit (Invitrogen), CD18 PECy5, CD66b PERCPCy5.5, CD14 BV421, HLA DR BV510, CD15 605, CD20 AF700, CD19 APC Cy7, HLA DM PE, CD3 PECF594, and HLA ABC (BD Biosciences). Relevant fluorescence minus one (FMO) controls and negative controls were used to identify positively stained populations. Lymphocytes, monocytes and granulocytes were identified by size forward scattered light (FSC)/ side scattered light (SC]) and then further phenotyped into live CD3− CD14+ monocytes, CD3+ lymphocytes, CD3− CD19/CD20 expressing B cells and CD66b/CD15 positive neutrophils. Flow cytometry was performed on the Fortessa instrument (BD Biosciences) and data analysed using ‘Flow Jo’ (Tree Star) software.

### Trypan blue exclusion

PBMCs were extracted from whole blood of two healthy adult ‘naïve’ donors (BCG non-immunised, PPD negative) and infected with *M. tuberculosis* (MOI PBMC: CFU, 1:1). Uninfected samples served as controls. All whole blood experiments were performed in triplicate for each time-point (0, 48, 72 and 96 h) and cell viability was determined by counting the numbers of viable cells using a basic light microscope. Duplicate counts for each time-point were undertaken by two researchers, samples blinded and the results averaged.

### RNA extraction, concentration and quality determination

RNA was extracted using the RNeasy and miRNeasy whole blood RNA extraction kit (Qiagen, Valencia CA) for mRNA and miRNA respectively according to the manufacturer’s protocol and stored at −80 °C. Extracted amounts of total RNA were assessed for quantity and quality, using the NanoDrop 1000 (PeqLab Biotechnologie, Germany). For additional quality and RNA-integrity checks, samples were run on the 2100 Bioanalyzer (Agilent Technologies, Inc.). Only samples with a RNA integrity number (RIN) of 7 or above were taken forward for further analysis.

### cDNA amplification and processing of Illumina coding RNA arrays

The Illumina TotalPrep RNA amplification kit (Applied Biosystems) was used to convert samples to biotin labelled cRNA that was directly hybridised to the Illumina HumanHT-12 v4 Beadchip (47,231 probes) (Illumina Inc. CA, USA) according to manufacturer’s procedures. After washing, blocking and staining, arrays were scanned using an Illumina Beadarray reader according to manufacturer’s instructions (Illumina Inc, CA, USA). Data was imported to Genome Studio software and the microarray images were inspected for artefacts and QC parameters were assessed. Mean raw intensity values for each probe were corrected for local background intensities and a robust spline normalisation (combining quantile normalisation and spline interpolation) was applied to each array.

### Labelling, hybridization and processing of Agilent non-coding RNA arrays

Total RNAs from whole blood samples were extracted using miRNeasy (QIAGEN). Labelling, microarray hybridization, washing, scanning and feature extraction were performed as in Dvinge *et al*.^48^. In brief, 100 ng RNA samples were labelled using Agilent miRNA Complete Labelling and Hyb Kit (p/n 5190-0456) and MicroRNA Spike-In Kit (p/n 5190-1934) without additional column-based purification. Labelled samples were hybridized to custom Agilent 8 × 60k arrays and washed using Gene Expression Wash Buffer Kit (p/n 5188–5327) following manufacturer’s recommendations with the following modifications: (a) prior to labelling RNA samples were vacuum-dried to completion at 45 °C; (b) hybridization volume was increased to 50 μL by addition of 1x Hi-RPM; (c) hybridization took place over 44 h. Microarrays were scanned on an Agilent Microarray Scanner (G2565CA) using miRNA_107_Sep09 scanning protocol (Agilent). Spot intensities were then extracted using Agilent’s Feature Extraction software v10.7.3 following the custom grid 029140_D_20100630_ns.

### Pre-processing of non-coding RNA array data

Pre-processing and normalization were performed as in Divinge *et al*.^48^. In brief, spot intensities were used without background correction. Outlying intra-array replicates (mostly due to physical imperfections) were removed iteratively by ranking the replicates of each spots (six for all except control probes) by intensity, removing the replicate furthest from the median, and repeating the ranking and removal once more, leaving four replicate spots for each miRNA probe. Data from multiple arrays was normalised using qspline and downstream analyses were carried out on median values of qspline-normalised data.

### Time-course microarray data analysis

We used a smoothing splines mixed-effects (SME) model^18^ to independently model each probe on the microarray and identify those exhibiting a significant change in expression levels over time. The SME model is a specific example of the functional mixed-effects model which has become popular in the analysis of replicated time course gene expression data^49–51^ due to its ability to handle missing observations, small sample sizes, few and irregularly spaced time points and subject heterogeneity that typifies such experiments. The resulting model for each probe can be summarised as a mean function (or analogously a curve) describing the average expression levels over time across all subjects, and individual curves for each subject representing their deviations from that mean. Probes showing a significant change in expression levels over time were identified by testing the null hypothesis that the mean curve is zero, implemented using a Wald test. A multiple testing correction was applied using the false discovery rate (FDR) method of Benjamini and Hochberg^52^. All time course analysis was carried out in R^53^ using the development version of the SME package available on GitHub (www.github.com/mberk/sme). Full details of the time-course analysis can be found in Supplementary Text S1.

### Biological pathway analysis

Significantly differentially expressed (SDE) genes were analysed through the use of Ingenuity Pathways Analysis (Ingenuity Systems®, www.ingenuity.com) to give the biological functions and pathways represented in the dataset. A Fisher’s exact test with a Benjamini-Hochberg multiple testing correction was used to test if there was an association between the SDE genes and the pathway/biological function, and whether this was due to chance alone. Pathways were also assessed using the ratio of the number of SDE genes that mapped to a pathway divided by the total number of genes that existed in the canonical pathway. Mapping of SDE genes to individual signalling pathways was performed using IPA® and KEGG pathways (http://www.genome.jp/kegg/pathway.html).

MicroRNA-mRNA association analysis was performed within *MicroRNA Target Filter* tool of IPA®. This feature uses experimentally validated interactions from TarBase and miRecords, as well as predicted miRNA-mRNA interactions from TargetScan. Additionally, IPA® includes a large number of miRNA-related findings from the peer-reviewed literature, the IPA® knowledge database.

SDE miRNAs (q-value < 0.001), both up- and down-regulated, were taken forward for analysis. In order to explore potential association between SDE mRNA transcripts and respective SDE miRNAs, all SDE mRNA transcripts (q-value < 0.01) were selected for correlation analysis using *MicroRNA Target Filter*. This analytic strategy was chosen to reflect the fact that an SDE miRNA, involved in the gene regulatory processes at the mRNA transcript level during whole blood infection with *M. tuberculosis*, could be either up- or down-regulated. Both findings would indicate an active involvement in the gene regulatory processes underway during the time course experiment. Lists of miRNA-mRNA relationships were filtered based on confidence level. Only “experimentally validated” and “highly probable” miRNA-mRNA target pairs were taken forward in the analysis. A hypergeometric test statistic was subsequently employed to calculate the significance of the respective association.

### Methylation arrays

Mycobacterial infection assays were performed with *M. tuberculosis* using whole blood taken from 5 healthy adult donors as described above. Uninfected whole blood samples from the same donors served as controls. All samples were incubated for 48 and 96 h at 37 °C as described. At each time-point DNA was extracted using the QIAamp whole blood midi kit (Qiagen) according to manufacturer’s protocols with one amendment: following the addition of buffer AL the sample was incubated for 4 h at 70 °C rather than 10 mins to ensure 100% mycobacterial killing before removing the samples from the Containment Level 3 laboratory. This procedure showed no detrimental effects to the quality or quantity of DNA extracted which was then measured using the fluorometric method (Qubit, Thermo Fisher Scientific, MA), spectrophotometry (Nanodrop, Thermo Fisher Scientific, MA) and Tapestation (Agilent Technologies, CA, USA). 500 ng of DNA was bisulfite-converted using EZ-DNA Methylation kit (Zymo Research, CA, USA) according to Illumina’s modified protocol. The bisulphite converted DNA was amplified, fragmented, precipitated and re-suspended and then hybridised to the Infinium HumanMethylation 450 beadarray (Illumina, CA) before extension and staining according to manufacturer’s instructions. Arrays were imaged on the Illumina iScan (Illumina, CA, USA). Data was quality controlled using GenomeStudio software (Illumina, CA, USA). We performed background correction on the raw intensity data according to Illumina guidelines to minimise technical variability, followed by exclusion of low quality array probes (detection p-value threshold of 10^−6^). The array probes were separated by colour channel (Red/Green), type (Type I/Type II) and subtype (M/U) and quantile normalisation of intensity values was applied^54,55^. We used the normalised intensities to calculate percentage methylation (β-values) at each marker and an empirical Bayes framework^56^ was used to mitigate batch effects, while adjusting for donor age and sex. Differential methylation analysis (Δbeta) at each locus, of individual markers between matched infected and uninfected samples at each time-point, was conducted using an empirical Bayes moderated t-statistic, which has been shown to have a more robust behaviour for smaller sample size experiments^57^. In addition we aggregated markers at the CpG-island level and at the gene level, to increase detection power by borrowing information across neighbouring markers. P-values were corrected for multiple testing using the Benjamini-Hochberg procedure^52^.

### Search for upstream regulatory elements

#### Sequences upstream of TSS

Using the Biomart webservice (http://www.biomart.org,^58^), probe names of significantly down- and up-regulated genes in response to infection of whole blood with *M. tuberculosis* were mapped into gene names and then filtered to include coding genes only; thus pseudogenes and other non-coding entities were excluded. Note that a number of probes could not be canonically mapped to genes or were mapped to genes in both the up- and down–regulated sets. These were removed from the analysis. This procedure was also followed for a set of “unchanged” genes that had demonstrated no significant differential regulation in the mycobacterial infection of whole blood dataset. Biomart was then used to download the 1,500 bp immediately upstream of the transcription start site (i.e. ATG codon) of each of these coding genes. This region was selected to examine only the immediately proximate elements. The number of sequences thus treated was 1,413 experimental (down-regulated) genes, 1,193 control (up-regulated genes) and 797 unchanged genes. Of the significantly down-regulated genes, a subgroup of 51 were identified as “phagosome-associated”, via pathway analysis as above. These were included as a biologically relevant subset of sequences for comparative analysis.

To ensure any shared motifs seen were not just the effect of examining duplicated and related genes, the coding regions of the down- and up-regulated genes were extracted from Biomart as described above and compared using the metagenomic tool usearch (http://www.drive5.com/usearch^59^). No matches above 50% were found.

#### Search for known regulatory elements

The upstream regions of down- and up-regulated genes were searched for known transcription factor binding sites (TFBS) using the OPOSSUM software (http://opossum.cisreg.ca/^23^). Upstream regions of the controls were used for background control purposes. No sites were found with better than Z-score of 10 or greater and a Fisher score of 7 or greater (limits suggested by tool authors from empirical studies).

#### Search for novel motifs

MEME^60^ was used to search more generally for conserved motifs in the 1,500 bp upstream region of the coding genes. The 100 top ranked down-regulated coding genes were used in this search, with 100 top up-regulated genes as the discriminative (control) gene set. The size of these subsets was used to limit computational demands. Based on previous experience, parameters were chosen to search for the 8 most significant motifs, allowing them to occur with any frequency, in either forward or reverse direction, i.e. on either strand. The motif size was limited to between 10–50 bases also for computational demands reasons. Several motifs, 26–36 bp in length, were identified. This procedure, and all subsequent steps, was automated with a computational pipeline (written in Snakemake^61^, available on request) for reproducibility and consistency (Fig. 6).

#### Motif cassette discovery and assembly

Examination of the above motif patterns showed many were occurring in regular repeating sequences, or “cassettes”. These cassettes were composed of motifs found on either strand or in either orientation. For example, a cassette might be *1*+*2*−*3*+: the first, second and third motifs found by MEME, occurring on the forward, reverse and forward strands in that order respectively. This cassette is complementary to *3*−*2*+*1−*. Note that MEME and our discovery process, does not determine which orientation is “correct” or which cassette is the most significant.

A modified general sequence pattern (GSP)^24,25^ algorithm (Fig. 5 and Supplementary Fig. S6) was used to recognize and enumerate these cassettes within the above MEME results. In brief, the basic algorithm generates an initial pool of patterns seen from the list of motifs seen. It then proposes longer potential patterns by merging every possible pairing of existing patterns that overlap. For example: the pattern *1*+*2*−*3*+ can be merged with *2*−*3*+*4−* to produce 1+*2*−*3*+*4−*. These proposed patterns are checked against the actual data and those that occur above a given cut-off frequency are retained with the next round of proposals. This merge-propose-filter cycle is repeated until no longer patterns can be found or the frequency of those found falls below the given cut-off. That is, GSP will find the longest pattern or patterns that occur above a specified frequency. A number of cassettes of varying lengths (2–8 motifs) were discovered.

This standard GSP algorithm was modified in a number of important ways. First, to find the frequencies of all cassettes, the GSP algorithm was repeated over a range of cut-off frequencies from 1% to 50% and the results combined. Second, cassettes were allowed to occur and be counted in either orientation. That is, when counting occurrences of *1*+*2*−*3*+, the complement *3*−*2+1−* would also be counted. Finally, before searching for patterns, insignificant motifs instances (i.e. those examples of a given motif falling above a significance threshold) were transformed into a null symbol. For example, if a sequence shows the motif pattern 1+2−3+, but the 2- instance is above the significance threshold, the sequence is transformed to 1+X3+, where X is the null symbol. These nulls are never used in building proposed patterns. Thus these insignificant motifs never appeared as part of a cassette but did serve to separate other motifs, breaking up potential patterns. For example, 1+X3− would never be matched by 1+2−, 2−3+, 1+2−3+ or 1+3+. A significance threshold of 1e-5 was used based on previous experience.

#### Cassette enumeration in sequence datasets

To enumerate the occurrence of these cassettes in other, non-discovery, sequence datasets, the MAST tool (part of MEME suite^62^) was used. This takes the motif patterns previously determined in the MEME results and searches for them in other sequences.

The sequence sets targeted were the remainder of the down- and up-regulated datasets (i.e. those not used for the initial discovery of the motifs, 1,312 and 1,093 respectively), the unchanged genes (797), and a comparative subset of the down-regulated sequences (51 phagosome-associated genes). The patterns for the component motifs found in any cassette were extracted and given to MAST to identify and locate motifs instances within these sequence datasets. After transforming insignificant motifs into null symbols as above, the resulting patterns of motifs were then mined for the cassette patterns detected above. The fraction of sequences with cassettes in each dataset was calculated, as well as the enrichment relative to the control (up-regulated) sequences. A p-value for this enrichment was calculated using the beta-binomial distribution. A table of the genes with the cassettes found upstream of them was produced for the phagosome-related genes.

#### Consensus & database search

“Exemplar” sequences for each cassette were obtained by identifying for each cassette the three best instances (i.e. those with the most significant p-value, calculated by combining the p-value of each constituent motif) and extracting the associated sequence. The respective cassette sequences were then interrogated using standard blastn searches (Supplementary Table S5). Initial BLAST was performed against the “Reference RNA sequences” (refseq_rna) tool and subsequently using the “Human *Alu* elements” tool within the blastn suite.

### Comparison of host transcriptomic data from *in vitroM. tuberculosis* infection with data derived from *in vitro* Leishmania infection

A comparative transcriptomic profiling analysis was undertaken of SDE genes from our *M. tuberculosis* whole blood dataset and respective host SDE genes in response to two Leishmania species from a study by Fernandes *et al*.^26^ to determine whether a similar pattern of host immune gene expression could be observed. Fernandes *et al*.^26^ performed transcriptomic profiling of human macrophages after infection with *L. major* or *L. amazonensis* over a 72 h time course. From the supplementary material of Fernandes *et al*.^26^ we obtained a list of the most SDE genes at each timepoint (4, 24, 48 and 72 h) along with the corresponding log_2_ FC. To facilitate comparison with our dataset, for each of the SDE genes we obtained the maximum log_2_ FC across all time points. We then compared the log_2_ FC of the most significant 1000 *M. tuberculosis* SDE genes against the log_2_FC of the same genes in both Leishmania infections.

### Data availability

Gene expresson data from this study is publicly available at the Gene Expression Omnibus (GEO) under accession number GSE108363 (https://www.ncbi.nlm.nih.gov/geo/query/acc.cgi?acc=GSE108363).

## Electronic supplementary material

Supplementary information - including Suppplementary Table 8

Supplementary Table 1

Supplementary Table 2

Supplementary Table 3

Supplementary Table 4

Supplementary Table 5

Supplementary Table 6

Supplementary Table 7

Supplementary Table 9

Supplementary Table 10
