# Supplementary material for: Mycobacterium tuberculosis Exploits a Molecular Off Switch of the Immune System for Intracellular Survival
Source: Sci Rep. 2018 Jan 12;8:661. doi: 10.1038/s41598-017-18528-y (PMC5766484; doi:10.1038/s41598-017-18528-y)
Supplement: Supplementary file 1 — Supplementary information - including Suppplementary Table 8 [file 41598_2017_18528_MOESM1_ESM.pdf]

Supplementary Information

Mycobacterium tuberculosis Exploits a Molecular *Off Switch* of the Immune System for Intracellular Survival

Ulrich von Both, Maurice Berk, Paul-Michael Agapow, Joseph D Wright, Anna Git, Melissa Shea Hamilton, Greg Goldgof, Nazneen Siddiqui, Evangelos Bellos, Victoria J Wright, Lachlan J Coin, Sandra M Newton, Michael Levin

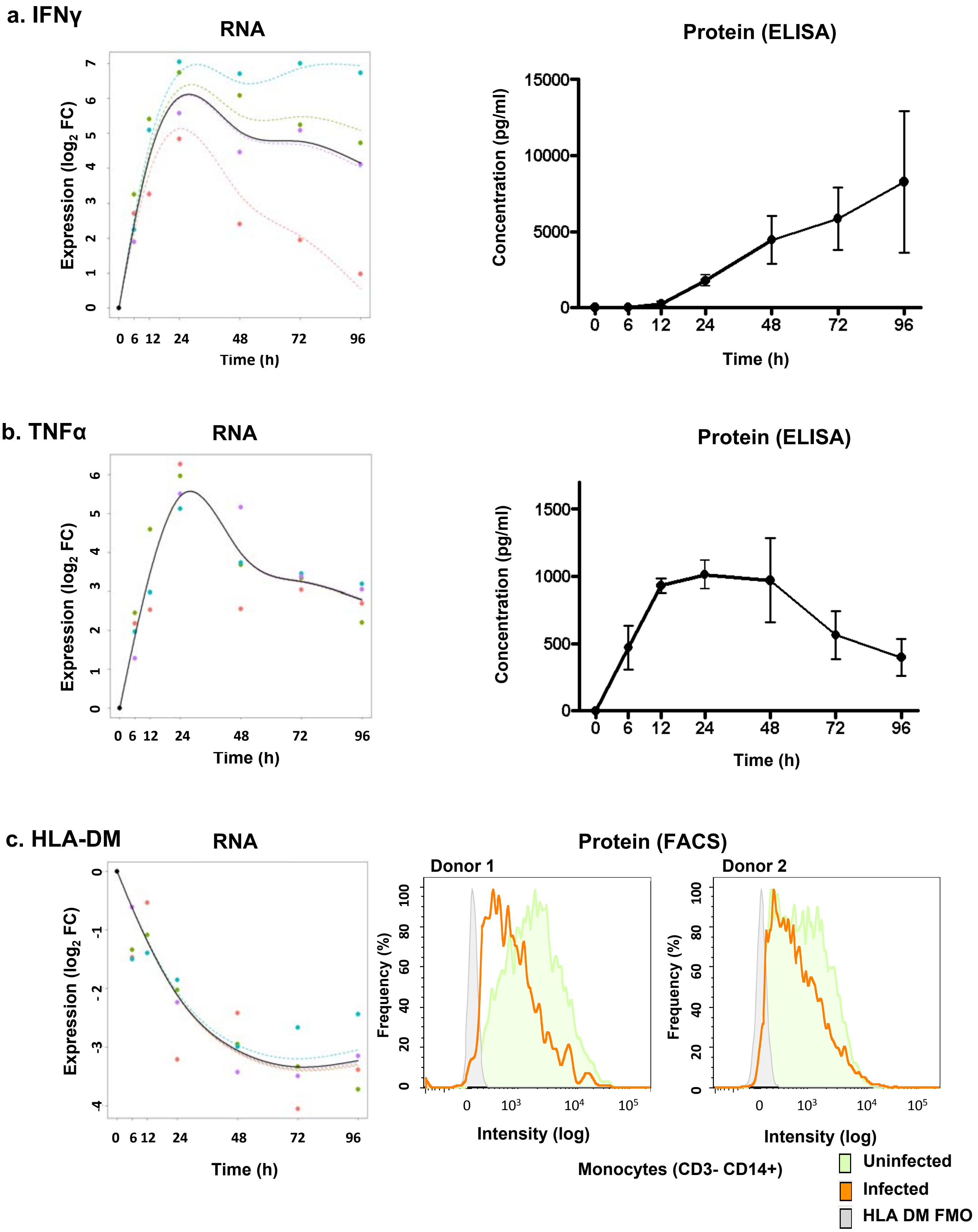

S1 Fig. mRNA expression correlates with protein measurements in *M. tuberculosis* infected whole blood
A smoothing mixed effects model was used to independently model each probe on the microarray and identify those exhibiting a significant change in expression levels over time. **a-c**: mRNA expression over time (0-96 h; n= 4, discovery set), in infected compared to uninfected matched samples, and detection over time of the corresponding directly secreted proteins, measured by ELISA, from the *M. tuberculosis* infected samples (n=4), TNF-α and IFN-γ (**a and b**) and by FACS for cell-associated protein HLA-DM on monocytes at 48 h (n =2) (**c**). Gene expression graphs: coloured dots represent gene expression (log_2 FC) for individual donors; black smooth lines represent mean gene expression levels over time across all donors and individual coloured curves correspond to each donor and represent their deviations from that mean. ELISA graphs: Each point represents the mean value of 4 donors, taken from triplicate samples at each time point for each blood donor. FACS graphs: FMO – Fluorescence Minus One Control.

a Early

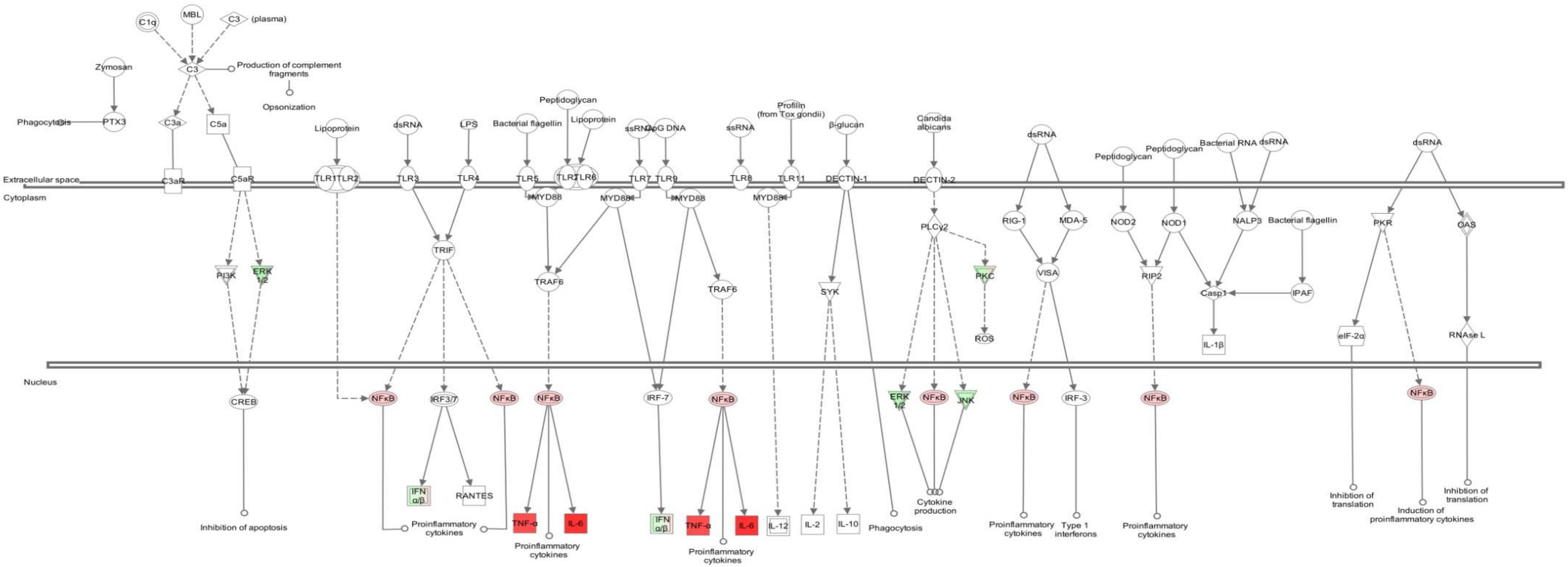

C1q
MBL
C3 (plasma)
Zymosan
C3
Production of complement fragments
Opsonization
Phagocytosis
PTX3
C3a
C5a
Peptidoglycan
Lipoprotein
Profilin (from Tox gondii)
Lipoprotein
dsRNA
LPS
Bacterial flagellin
ssRNA
CpG DNA
ssRNA
β-glucan
Candida albicans
dsRNA
Peptidoglycan
Peptidoglycan
Bacterial RNA
dsRNA
dsRNA
Extracellular space
Cytoplasm
C3aR
C5aR
TLR1
TLR2
TLR3
TLR4
TLR5
TLR2
TLR6
TLR7
TLR9
TLR8
TLR11
DECTIN-1
DECTIN-2
MYD88
MYD88
MYD88
MYD88
PLCγ2
RIG-1
MDA-5
NOD2
NOD1
NALP3
Bacterial flagellin
PKR
OAS
TRIF
PI3K
ERK 1/2
TRAF6
TRAF6
SYK
PKC
VISA
RIP2
Casp1
IPAF
ROS
IL-1β
eIF-2α
RNAse L
Nucleus
CREB
NFκB
IRF3/7
NFκB
NFκB
IRF-7
NFκB
ERK 1/2
NFκB
JNK
NFκB
IRF-3
NFκB
NFκB
IFN α/β
RANTES
TNF-α
IL-6
IFN α/β
TNF-α
IL-6
IL-12
IL-2
IL-10
Inhibition of apoptosis
Proinflammatory cytokines
Proinflammatory cytokines
Proinflammatory cytokines
Phagocytosis
Cytokine production
Proinflammatory cytokines
Type 1 interferons
Proinflammatory cytokines
Inhibition of translation
Induction of proinflammatory cytokines
Inhibtion of translation

b Late

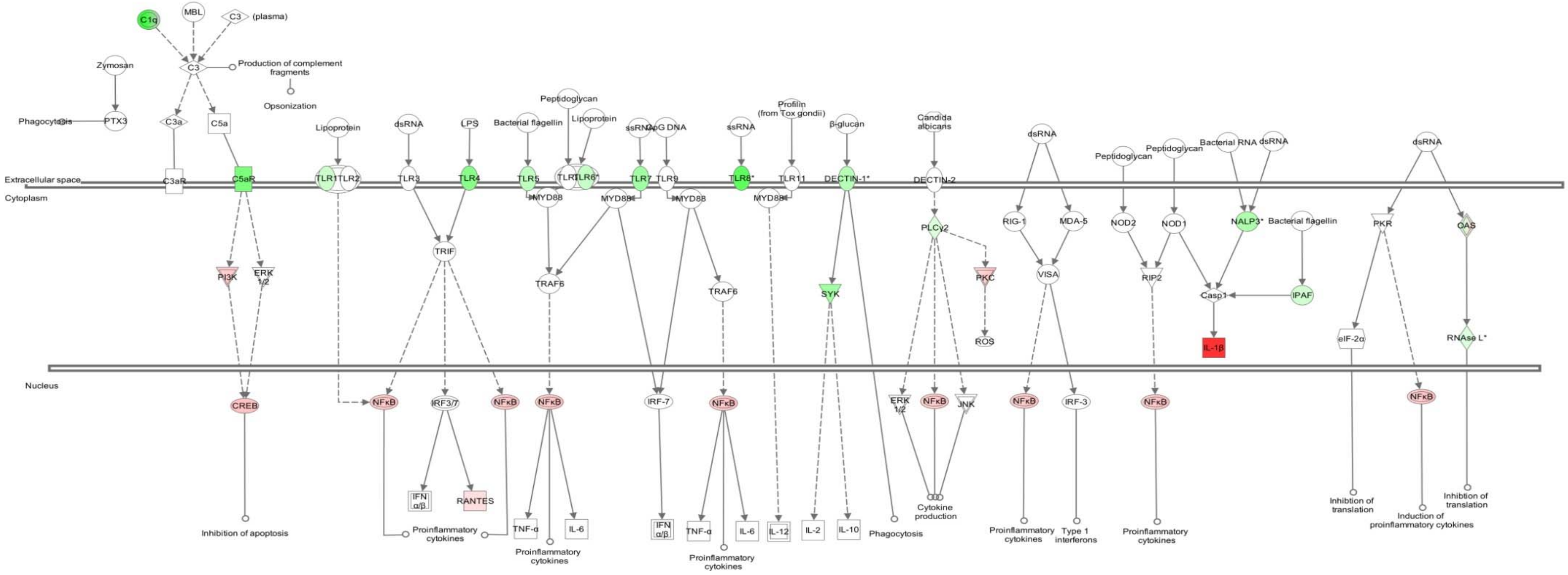

S2 Fig. Dynamic changes in pattern recognition receptor signalling pathway in response to *M. tuberculosis* infection of whole blood

Significantly differentially expressed (SDE) genes were analysed through the use of Ingenuity Pathways Analysis (IPA®) to give the biological functions and pathways represented in the dataset.

a. Early response (genes reaching |max. $\log_2$FC| within 48 h of infection).

b. Late response (genes reaching |max. $\log_2$FC| between 48 and 96 h of infection).

SDE genes are coloured in red (up-regulated) and green (down-regulated). Intensity of colour indicates degree of abundance of each transcript

a Early

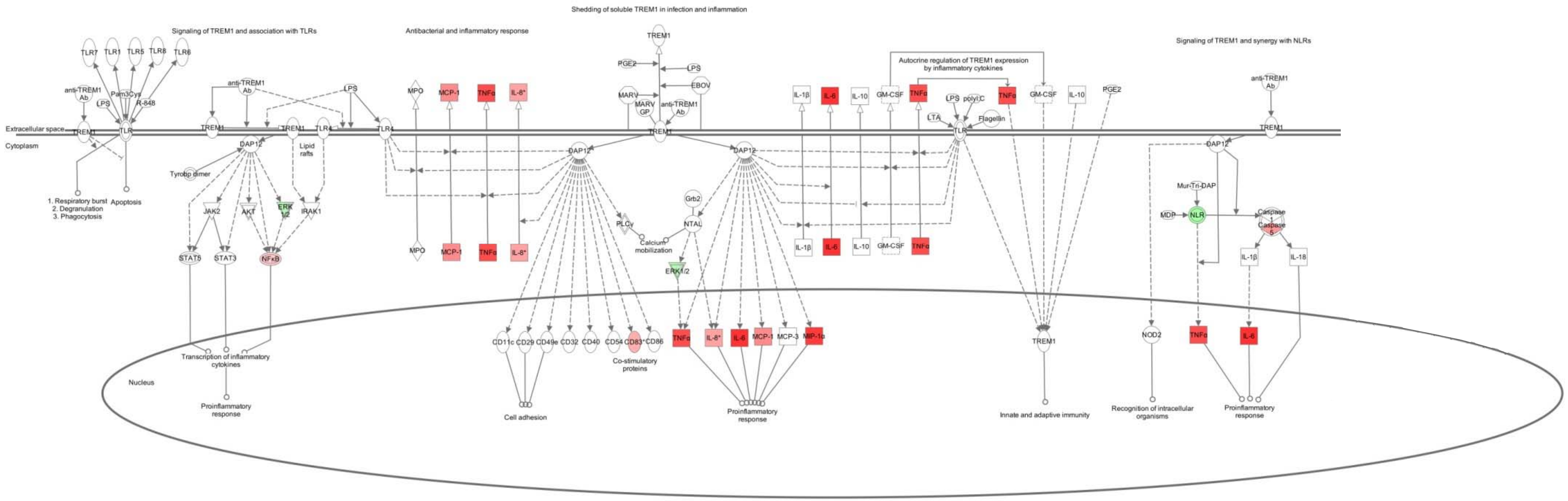

Shedding of soluble TREM1 in infection and inflammation
Signaling of TREM1 and association with TLRs
Antibacterial and inflammatory response
Signaling of TREM1 and synergy with NLRs
Autocrine regulation of TREM1 expression by inflammatory cytokines
Extracellular space
Cytoplasm
Nucleus
TLR7
TLR1
TLR5
TLR8
TLR6
anti-TREM1 Ab
Pam3Cys
LPS
R-848
TREM1
TLR
TLR4
DAP12
Lipid rafts
Tyrobp dimer
JAK2
AKT
ERK1/2
IRAK1
STAT5
STAT3
NFκB
1. Respiratory burst
2. Degranulation
3. Phagocytosis
Apoptosis
Transcription of inflammatory cytokines
Proinflammatory response
MPO
MCP-1
TNFα
IL-8*
PGE2
EBOV
MARV
MARV GP
Grb2
NTAL
PLCγ
Calcium mobilization
CD11c
CD29
CD49e
CD32
CD40
CD54
CD83*
CD86
Co-stimulatory proteins
Cell adhesion
IL-6
MCP-3
MIP-1α
IL-1β
IL-10
GM-CSF
polyI:C
LTA
Flagellin
Innate and adaptive immunity
Mur-Tri-DAP
MDP
NLR
Caspase 1
Caspase 5
IL-18
NOD2
Recognition of intracellular organisms

b Late

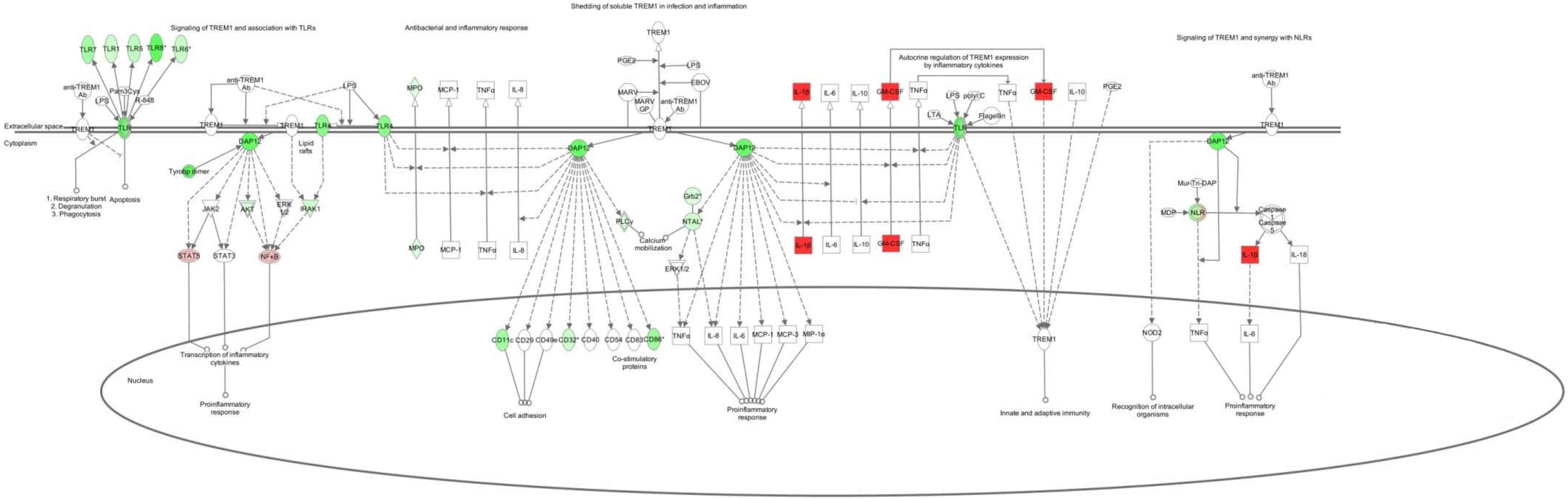

S3 Fig. Dynamic changes in TREM1 signalling pathway in response to *M. tuberculosis* infection of whole blood

Significantly differentially expressed (SDE) genes were analysed through the use of Ingenuity Pathways Analysis (IPA®) to give the biological functions and pathways represented in the dataset.

a. Early response (genes reaching |max. $\log_2$FC| within 48 h of infection).

b. Late response (genes reaching |max. $\log_2$FC| between 48 and 96 h of infection).

SDE genes are coloured in red (up-regulated) and green (down-regulated). Intensity of colour indicates degree of abundance of each transcript.

a b

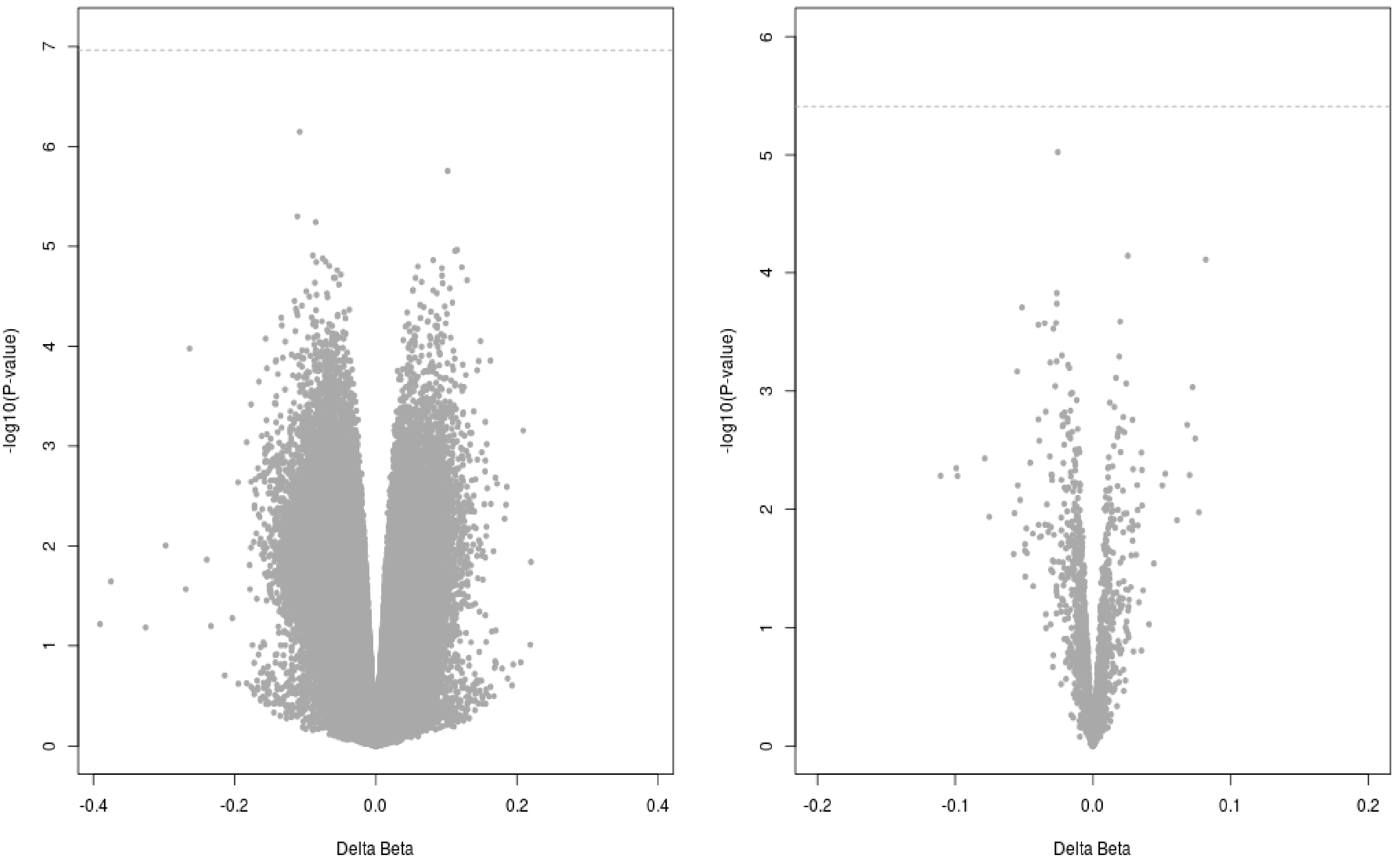

S4 Fig. Differential DNA methylation was not associated with the changes in gene expression in *M. tuberculosis* infected whole blood at 96 h (*n*=5).

a. Volcano plot showing changes in methylation across all individual methylation markers on the Illumina Infinium Human Methylation 450 beadarrays array.

b. Volcano plot showing methylation changes across genes. This was achieved by aggregating markers within 1kb of each gene. Methylation differences (delta beta) are represented on the x-axis, while *p*-values are represented on the y-axis on a logarithmic scale. Dashed lines denote the epigenome-wide significance threshold.

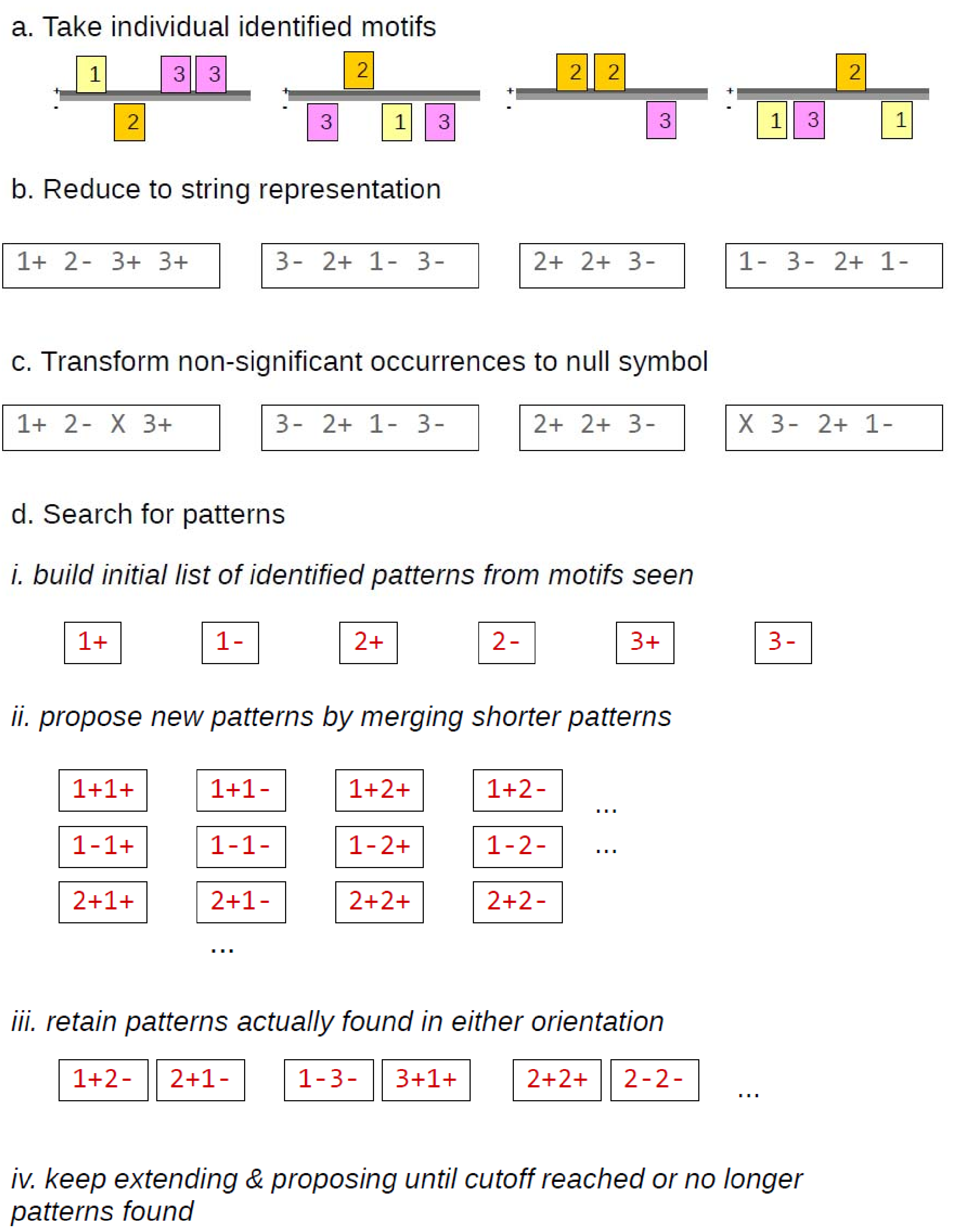

S5 Fig. The modified General Sequence Pattern (GSP) algorithm

A modified GSP algorithm was used to recognize and enumerate the cassettes.

a. Discover the pattern of motifs in the sequence data. **b.** Reduce this pattern to a string representation. **c.** Transform all non-significant motifs to a null symbol ('X'). **d.** **i)** Start the GSP algorithm with an initial pool of found patterns composed of all motifs discovered, but not including the null symbol. **ii)** Propose new and longer patterns by merging all compatible pairs of existing patterns **iii)** Retain patterns that are actually seen **iv)** Repeat until no extension is possible.

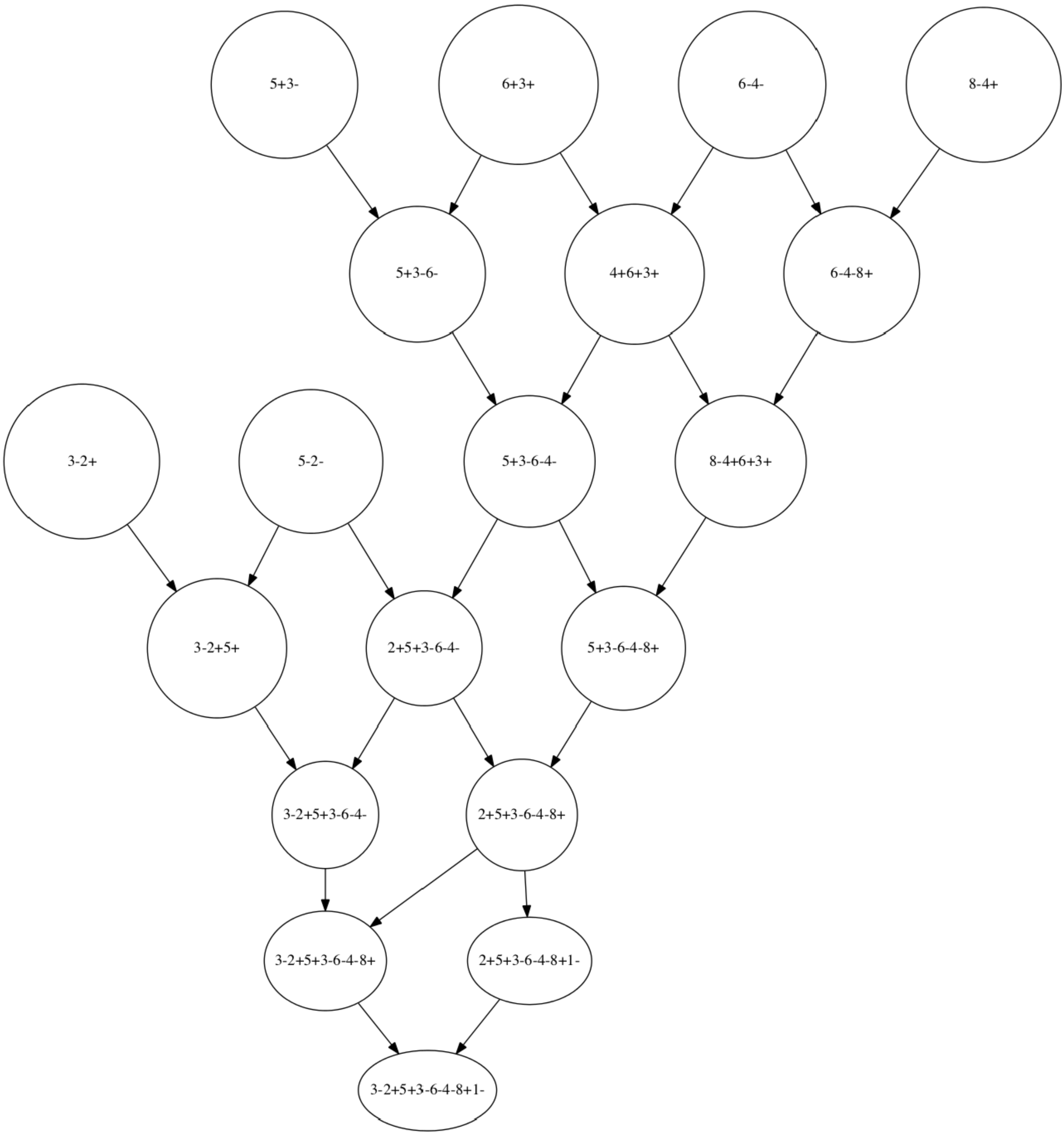

S6 Fig. Discovery and relationship of cassettes identified from the motif patterns using the General Sequence Pattern (GSP) algorithm

Examination of the upstream 1500bp region of the significantly differentially expressed (SDE) down-regulated genes compared to the SDE up-regulated genes from *M. tuberculosis* infection of whole blood over 96 h, identified a series of motifs shown to combine to form cassettes using the GSP algorithm. Node height is proportional to frequency. The "tree" arrangement shows which shorter cassettes fall within longer cassettes but does not imply any evolutionary or other arrangement.

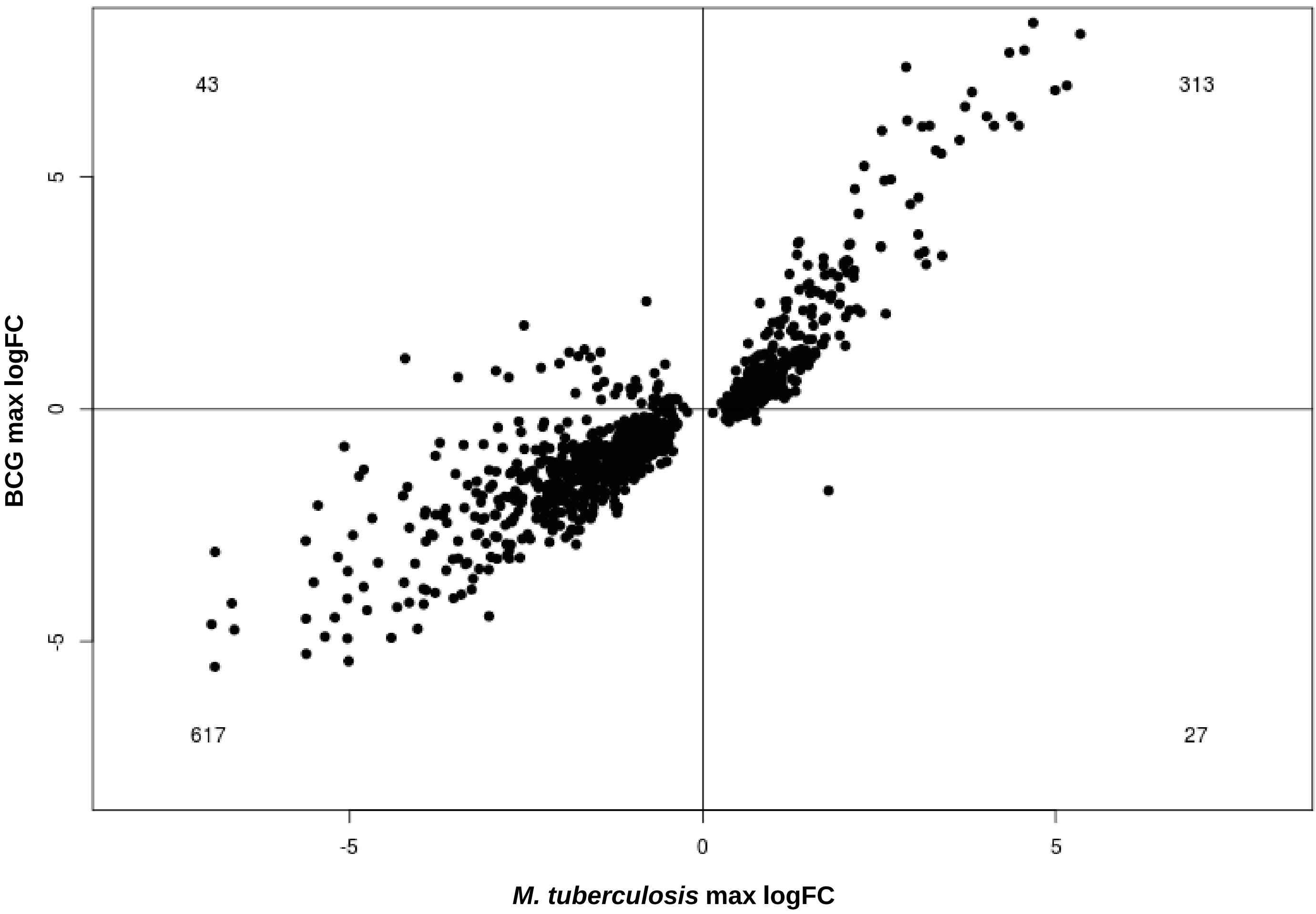

S7 Fig. Gene expression in whole blood infected with *M. tuberculosis* is highly correlated with that of *M. bovis* BCG infection.

Correlation of |maximum logFC| between SDE transcripts in whole blood infected with *M. tuberculosis* (top 1000 SDE probes from *M. tuberculosis* dataset) compared to *M. bovis* BCG. 88% of genes showing significant differential expression in response to *M. tuberculosis* infection, did follow the same direction of gene regulation (up or down) when whole blood was infected with *M. bovis* BCG (p-value 2.2e-16).

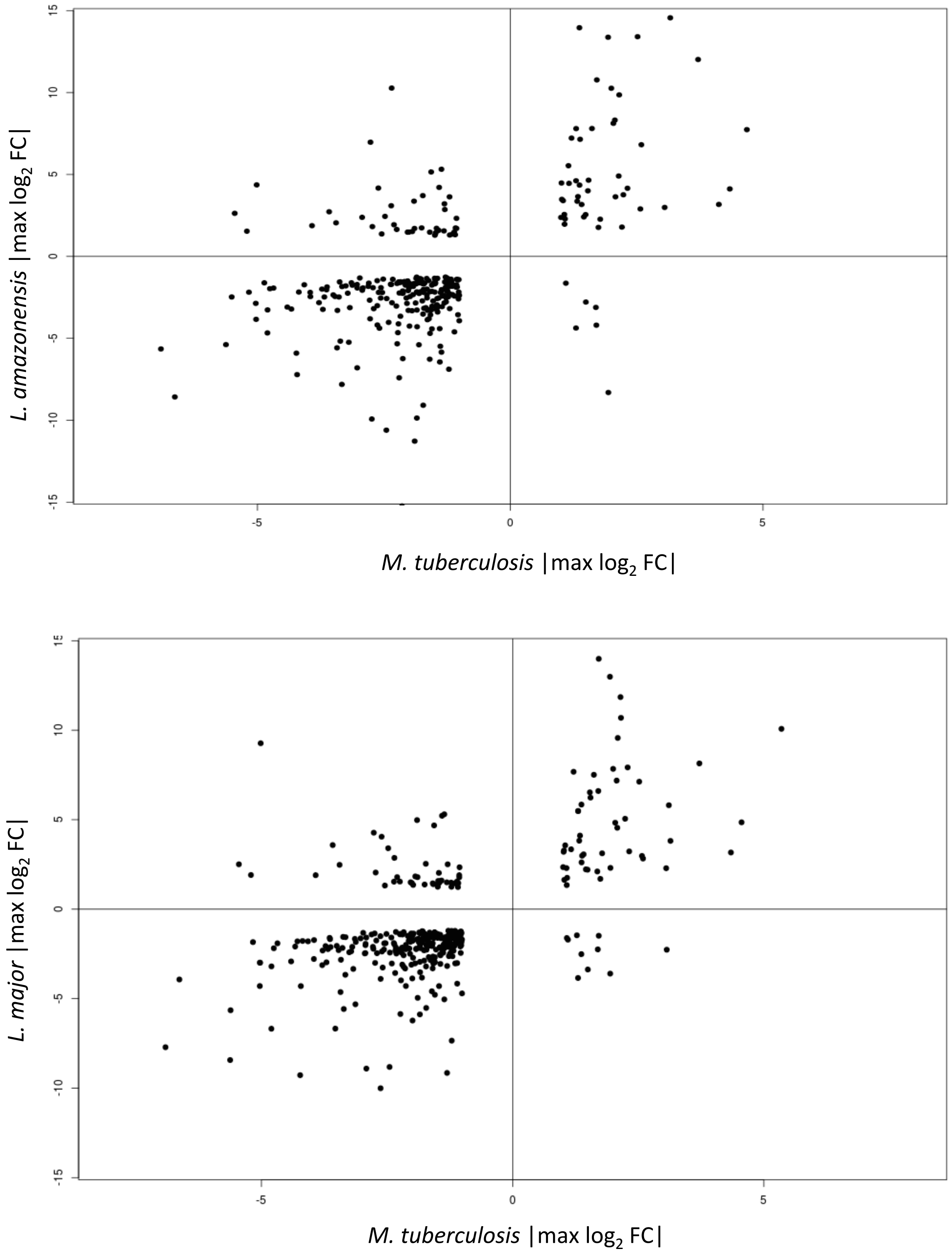

S8 Fig. Differences in gene expression in *M. tuberculosis* infected whole blood compared to both *L. amazonensis and L. major infected* human macrophages.

Correlation of |maximum log_2FC| between SDE transcripts in whole blood infected with *M. tuberculosis* for 96 hours (top 1000 SDE probes from *M. tuberculosis* dataset) with significantly differentially expressed transcripts in human macrophages infected with either *L. amazonensis* (top) or *L. major* (bottom) over a 72 h time course (*Leishmania* data derived from Fernandes *et al.* Mbio 2016) . A significant proportion of SDE transcripts in response to *M. tuberculosis* infection of whole blood followed the same direction of gene regulation (up or down) as SDE transcripts derived from *in vitro* infection of human macrophages with *L. amazonensis,* r=60.5%, (95% CI: (53.2%, 66.9%), p value=2.2e-16) and *L. major*, r=56.6%, 95% CI: (49.3%, 63.2%), p value=2.2e-16. r = Pearsons correlation coefficient.

Identity matches to *ALU* sequences	Cassette
100% to ALUS(x) and others	*3+5-*
100% to ALUS(x) and others	*3-2+*
94% to ALUS(x) and others	*3-6-*
100% to ALUS(x) and others, like AluSb	*4+6+*
98% to ALUS(x) and others, like AluSb	*4-8+*
94% to ALUS(x) and others, 98% to AluSb	*5-2-*
100% to ALUSb 95% to AluSx	*3-2+5+*
92% to ALUS(x) and others, like AluSb	*3-6-4-*
99% to ALUSq and others, 98% to AluSx	*6+3+5-*
98% to ALUS(x) and 97% to AluSb	*8-4+6+*
97% to ALUS(x) and ALUSb	*3-6-4-8+*
92% to ALUS(x)	*4+6+3+5-*
92% to ALUS(x)	*4+6+3+5-2-*
92% to ALUS(x), 94% ALUSp	*8-4+6+3+5-*
99% to ALUSb, 92% to AULSx	*4+6+3+5-2-3+*
99% to ALUSb, 94% to AULSx	*8-4+6+3+5-2-*
94% to ALUS(x)	*1+8-4+6+3+5-2-*
99% to ALUSb, 95% to AULSx	*8-4+6+3+5-2-3+*
98% to ALUSb and 94% ALUSx	*1+8-4+6+3+5-2-3+*

S8 Table. ‘Exemplar’ cassette sequences match to *Alu* regulatory elements
A BLAST of the respective cassette sequences was performed against the “Reference RNA sequences” (refseq_rna) tool and the “human *Alu* repeat elements” (*Alu* repeats) tool within the blastn suite.

Supplementary text 1 – Analysis of Microarray Time-course Data see text script below

S1 Text. Details of novel script used for time-course analysis
Technical details for (i) the smoothing splines mixed-effects (SME) model used to fit the microarray time course data and (ii) the Wald type test applied to the fitted SME models to detect significantly differentially expressed probes.

1 Analysis of Microarray Time Course Data

We fit each probe on the microarray using a smoothing splines mixed-effects (SME) model (Berk et al. 2011). The SME model is a specific example of the functional mixed-effects model which has become popular in the analysis of replicated time course gene expression data (Storey et al. 2005; Liu and Yang 2009; Berk et al. 2010) due to its ability to handle missing observations, small sample sizes, few and irregularly spaced time points and subject heterogeneity that typifies such experiments.

Under the SME model, it is assumed that the observations on subject i have arisen from some underlying smooth function of time, $y_i(t)$, which can be decomposed into the following components:

$$y_i(t) = \mu(t) + v_i(t) + \epsilon_i(t) \tag{1}$$

where $\mu(t)$ is a mean function of time across all subjects, $v_i(t)$ is subject i's deviation from that mean function, also assumed to be a smooth function of time, and $\epsilon_i(t)$ is an error process. Analogous to the standard mixed-effects model, $\mu(t)$ is assumed to be some fixed population parameter while the $v_i(t)$ are assumed to be randomly sampled from the population as a whole. Typically, the $v_i(t)$ are assumed to be independent realisations of a Gaussian Process with zero mean and covariance function $\gamma(s,t)$ (Wu and Zhang 2006).

In the SME model the functions $\mu(t)$ and $v_i(t)$, $i = 1, \cdots, n$ are represented as smoothing splines. To begin, (1) is written in matrix-vector format as:

$$\boldsymbol{y}_i = \boldsymbol{X}_i\boldsymbol{\mu} + \boldsymbol{X}_i\boldsymbol{v}_i + \boldsymbol{\epsilon}_i \tag{2}$$

where $\boldsymbol{y}_i$ is an N_i length vector of all observations collected on subject i, $\boldsymbol{X}_i$ is a known *incidence* matrix of dimension $N_i \times p$ where p is the number of distinct *design* time points across all subjects, $\boldsymbol{\mu}$ is a vector containing the values of $\mu(t)$ evaluated at the design time points and similarly for $\boldsymbol{v}_i$, and $\boldsymbol{\epsilon}_i$ is an N_i length vector of all error terms corresponding to the observations on subject i.

The incidence matrix $\boldsymbol{X}_i$ maps the design time points onto the time points at which subject i was actually observed. This is in order to cater for two situations: (1) when data is missing, either due to the experimental design or due to errors in the measurement process such that subject i is not observed at all of the design time points and (2) when multiple observations on the same subject are available for a given time point due to technical replication. $\boldsymbol{X}_i$ is constructed in the following way: the jth row, corresponding to the jth observation on subject i, taken at time t_{ij}, contains zeroes in every column aside from the one corresponding to t_{ij} which contains a one.

(2) is in the form of the standard linear mixed-effects model (Harville 1977). Standard practice is to assume that the randomly sampled $\boldsymbol{v}_i$ are i.i.d. multivariate normal with zero mean and covariance matrix $\boldsymbol{D}$. Independently, the error terms $\boldsymbol{\epsilon}_i$ are assumed to be i.i.d. multivariate normal with zero mean and covariance matrix $\boldsymbol{R}$ although typically this is simplified to $\boldsymbol{R} = \sigma^2\boldsymbol{I}$. Under these assumptions, $\boldsymbol{y}_i$ is itself multivariate normally distributed with mean vector $\boldsymbol{X}_i\boldsymbol{\mu}$ and covariance matrix $\boldsymbol{V}_i = \boldsymbol{X}_i\boldsymbol{D}\boldsymbol{X}_i^T + \sigma^2\boldsymbol{I}$. The model parameters $\boldsymbol{\mu}$, $\boldsymbol{D}$ and σ^2 can then be estimated via maximum likelihood either by treating the random effects $\boldsymbol{v}_i$ as missing data and employing the Expectation-Maximisation algorithm (Laird and Ware 1982), or through direct maximisation (Lindstrom and Bates 1988).

In the SME model, however, as the functions $\mu(t)$ and $v_i(t)$ are represented as smoothing splines, it is instead necessary to estimate the model parameters by maximising the *penalised* likelihood. This is the same as the standard likelihood with the addition of penalty terms for the *roughness* of the functions. Usually this roughness is quantified as the integral of the squared-second derivative (Ramsay and Silverman 2005).

The penalised complete log-likelihood across all subjects can then be written as:

$$\log f(\boldsymbol{y}, \boldsymbol{v}|\boldsymbol{D}, \sigma^2, \boldsymbol{\mu}) - \lambda_\mu^* \int_a^b [\mu''(t)]^2 \, dt - \lambda_v^* \sum_{i=1}^n \int_a^b [v_i''(t)]^2 \, dt =$$

$$-\frac{N}{2}\log 2\pi - \frac{N}{2}\log\sigma^2 - \frac{1}{2\sigma^2}\sum_{i=1}^n ||\boldsymbol{y}_i - \boldsymbol{X}_i\boldsymbol{\mu} - \boldsymbol{X}_i\boldsymbol{v}_i||^2 - \lambda_\mu^* \int_a^b [\mu''(t)]^2 \, dt -$$

$$\frac{np}{2}\log 2\pi - \frac{n}{2}\log|\boldsymbol{D}| - \frac{1}{2}\sum_{i=1}^n \boldsymbol{v}_i^T \boldsymbol{D}^{-1}\boldsymbol{v}_i - \lambda_v^* \sum_{i=1}^n \int_a^b [v_i''(t)]^2 \, dt$$

where $\boldsymbol{y}$ is an $N = \sum_{i=1}^n N_i$ length vector formed by concatenating all of the $\boldsymbol{y}_i$ vectors and similarly for $\boldsymbol{v}$, and λ_μ^* and λ_v^* are *smoothing parameters* controlling the roughness of the fit. These smoothing parameters are non-negative real values which allow for a full spectrum of non-linear behaviours to be considered. When zero, the penalty term is non-existant and the functions can interpolate the data points (assuming no technical replication). As the smoothing parameters tend to infinity, the penalty term dominates, leading to a linear fit as the second derivative will then be zero. Note that, as presented here, the same smoothing parameter λ_v^* is used for all subject specific functions. In principle, a separate parameter per subject specific function could be used, at the expense of computational cost. However, the idea of a common smoothing parameter is conceptually sound, given the assumption that the subject specific functions arise from the same underlying Gaussian Process (Wu and Zhang 2006).

For the purposes of calculating the penalty terms, Green and Silverman (1994) conveniently show that for the case of cubic smoothing splines there exists a *roughness matrix* $\boldsymbol{G}$ such that:

$$\lambda_\mu^* \int_a^b [\mu''(t)]^2 \, dt = \lambda_\mu^* \boldsymbol{\mu}^T \boldsymbol{G}\boldsymbol{\mu}$$

$$\lambda_v^* \sum_{i=1}^n \int_a^b [v_i''(t)]^2 \, dt = \lambda_v^* \sum_{i=1}^n \boldsymbol{v}_i^T \boldsymbol{G}\boldsymbol{v}_i$$

Let the p distinct design time points be denoted as $\tau_1, \cdots, \tau_p$. Then the roughness matrix is given as $\boldsymbol{G} = \boldsymbol{A}\boldsymbol{B}^{-1}\boldsymbol{A}^T$ where the matrices $\boldsymbol{A}$ and $\boldsymbol{B}$ are derived as follows. Let $h_r = \tau_{r+1} - \tau_r$, $r = 1, \cdots, p-1$ denote the differences between sucessive time points. Then the matirx $\boldsymbol{A}$ is a $p \times (p-2)$ matrix whose entries $a_{r,s}$ are given by:

$$a_{r,r} = h_r^{-1} \quad a_{r+1,r} = -(h_r^{-1} + h_{r+1}^{-1}) \quad a_{r+2,r} = h_{r+1}^{-1}$$

for $r = 1, \cdots, p-2$ and 0 elsewhere. $\boldsymbol{B}$ is a $(p-2) \times (p-2)$ matrix with entries given by:

$$b_{1,1} = \frac{h_1 + h_2}{3} \quad b_{2,1} = \frac{h_2}{6}$$

$$b_{r,r+1} = \frac{h_{r+1}}{6} \quad b_{r+1,r+1} = \frac{h_{r+1} + h_{r+2}}{3} \quad b_{r+2,r+1} = \frac{h_{r+2}}{6} \quad r = 1, \cdots, p-4$$

$$b_{p-3,p-2} = \frac{h_{p-2}}{6} \quad b_{p-2,p-2} = \frac{h_{p-2} + h_{p-1}}{3}$$

Wu and Zhang (2006) suggest to incorporate the penalty terms more tightly into the penalised log-likelihood as follows. Let $\frac{\lambda_\mu}{2} = \lambda_\mu^*$ and $\frac{\lambda_v}{2} = \lambda_v^*$. Combined with the roughness matrix representation, this allows the

penalised complete log-likelihood to be written as

$$\log f(\boldsymbol{y},\boldsymbol{v}|\boldsymbol{D},\sigma^2,\boldsymbol{\mu}) - \lambda_\mu^* \int_a^b \left[\mu''(t)\right]^2 dt - \lambda_v^* \sum_{i=1}^n \int_a^b \left[v_i''(t)\right]^2 dt =$$

$$-\frac{N}{2}\log 2\pi - \frac{N}{2}\log\sigma^2 - \frac{1}{2\sigma^2}\sum_{i=1}^n ||\boldsymbol{y}_i - \boldsymbol{X}_i\boldsymbol{\mu} - \boldsymbol{X}_i\boldsymbol{v}_i||^2 - \frac{\lambda_\mu}{2}\boldsymbol{\mu}^T\boldsymbol{G}\boldsymbol{\mu} -$$

$$\frac{np}{2}\log 2\pi - \frac{n}{2}\log|\boldsymbol{D}| - \frac{1}{2}\sum_{i=1}^n \boldsymbol{v}_i^T\boldsymbol{D}^{-1}\boldsymbol{v}_i - \frac{\lambda_v}{2}\sum_{i=1}^n \boldsymbol{v}_i^T\boldsymbol{G}\boldsymbol{v}_i$$

This format suggests that penalised maximum likelihood estimation may be facilitated by refining the initial distributional assumptions such that the covariance matrix of the random effects $\boldsymbol{v}_i$ is instead $\widetilde{\boldsymbol{D}} = (\boldsymbol{D}^{-1} + \lambda_v\boldsymbol{G})^{-1}$. Wu and Zhang (2006) term $\widetilde{\boldsymbol{D}}$ the *regualrised* covariance matrix of the random effects. Under these new distributional assumptions, the (regularised) covariance matrix of the observations $\boldsymbol{y}_i$ is now $\widetilde{\boldsymbol{V}}_i = \boldsymbol{X}_i\widetilde{\boldsymbol{D}}\boldsymbol{X}_i^T + \sigma^2\boldsymbol{I}$.

2 Detecting Differentially Expressed Probes

After fitting each probe with the SME model to obtain model parameters $\widehat{\boldsymbol{\mu}}$, $\widehat{\boldsymbol{D}}$ and $\widehat{\sigma}^2$, we proceed to identify those probes with significant changes in expression levels over time by testing the null hypothesis that $\boldsymbol{\mu} = \boldsymbol{0}$. Specifically, we construct the test statistic

$$W = \widehat{\boldsymbol{\mu}}^T \left[\mathrm{Cov}(\widehat{\boldsymbol{\mu}})\right]^{-1} \widehat{\boldsymbol{\mu}}$$

Under the distributional assumptions of the SME model

$$\mathrm{Cov}(\widehat{\boldsymbol{\mu}}) = (\boldsymbol{X}\widetilde{\boldsymbol{V}}^{-1}\boldsymbol{X} + \lambda\boldsymbol{G})^{-1}\boldsymbol{X}\widetilde{\boldsymbol{V}}^{-1}\boldsymbol{X}(\boldsymbol{X}\widetilde{\boldsymbol{V}}^{-1}\boldsymbol{X} + \lambda\boldsymbol{G})^{-1}$$

and

$$W \sim \chi^2_M$$

where M is the number of distinct time points.

Calculation of $\mathrm{Cov}(\widehat{\boldsymbol{\mu}})$ depends on the true values of $\boldsymbol{D}$ and σ^2 which are obviously unknown. Instead, we replace them with their estimates $\widehat{\boldsymbol{D}}$ and $\widehat{\sigma}^2$.

References

M. Berk, M. Levin, C. Hemingway, and G. Montana. Longitudinal analysis of gene expression profiles using functional mixed-effects models. In *Studies in Theoretical and Applied Statistics*, 2010.

M. Berk, T. Ebbels, and G. Montana. A statistical framework for metabolic profiling using longitudinal data. *Bioinformatics*, 27:1979 – 1985, 2011.

P. J. Green and B. W. Silverman. *Nonparametric Regression and Generalized Linear Models.* Chapman and Hall, 1994.

D. A. Harville. Maximum likelihood approaches to variance component estimation and to related problems. *Journal of the American Statistical Association*, 72(358):320–388, 1977.

N. M. Laird and J. H. Ware. Random-effects models for longitudinal data. *Biometrics*, 38:963–974, 1982.

M. J. Lindstrom and D. M. Bates. Newton-Raphson and EM Algorithms for Linear Mixed-Effects Models for Repeated-Measures Data. *Journal of the American Statistical Association*, 83(404):pp. 1014–1022, 1988.

X. Liu and M. C. K. Yang. Identifying temporally differentially expressed genes through functional principal components analysis. *Biostatistics*, page kxp022, 2009.

J. Ramsay and B. W. Silverman. *Functional Data Analysis.* Springer, New York, 2 edition, 2005.

J. D. Storey, W. Xiao, J. T. Leek, R. G. Tompkins, and R. W. Davis. Significance analysis of time course microarray experiments. *Proceedings of the National Academy of Sciences of the United States of America*, 102(36):12837–12842, Sep 2005.

H. Wu and J.-T. Zhang. *Nonparametric Regression Methods for Longitudinal Data Analysis.* Wiley, 2006. doi: 10.1002/0470009675.
